# Exophilone, a Tetrahydrocarbazol-1-one Analogue with Anti-Pulmonary Fibrosis Activity from the Deep-Sea Fungus *Exophiala oligosperma* MCCC 3A01264

**DOI:** 10.3390/md20070448

**Published:** 2022-07-09

**Authors:** Ming-Jun Hong, Meng-Jiao Hao, Guang-Yu Zhang, Hou-Jin Li, Zong-Ze Shao, Xiu-Pian Liu, Wen-Zhe Ma, Jun Xu, Taifo Mahmud, Wen-Jian Lan

**Affiliations:** 1 School of Pharmaceutical Sciences, Sun Yat-sen University, Guangzhou 510006, China; hongmj5@mail2.sysu.edu.cn (M.-J.H.); haomj@mail2.sysu.edu.cn (M.-J.H.); zhanggy39@mail2.sysu.edu.cn (G.-Y.Z.); xujun9@mail.sysu.edu.cn (J.X.); 2 School of Chemistry, Sun Yat-sen University, Guangzhou 510006, China; ceslhj@mail.sysu.edu.cn; 3 Key Laboratory of Marine Biogenetic Resources, Third Institute of Oceanography, Ministry of Natural Resources, Xiamen 361005, China; shaozz@163.com (Z.-Z.S.); mccc5177@163.com (X.-P.L.); 4 State Key Laboratory of Quality Research in Chinese Medicine, Macau University of Science and Technology, Taipa 519020, Macau, China; wzma@must.edu.mo; 5 Department of Pharmaceutical Sciences, Oregon State University, Corvallis, OR 97331, USA; Taifo.Mahmud@oregonstate.edu

**Keywords:** *Exophiala oligosperma*, marine fungus, pulmonary fibrosis (PF), molecular docking

## Abstract

A new compound, exophilone (**1**), together with nine known compounds (**2**–**10**), were isolated from a deep-sea-derived fungus, *Exophiala oligosperma*. Their chemical structures, including the absolute configuration of **1,** were elucidated using nuclear magnetic resonance (NMR) spectroscopy, high-resolution electrospray ionization mass spectroscopy (HRESIMS), and electronic circular dichroism (ECD) calculation. Compounds were preliminarily screened for their ability to inhibit collagen accumulation. Compounds **1**, **4**, and **7** showed weaker inhibition of TGF-β1-induced total collagen accumulation in compared with pirfenidone (73.14% inhibition rate). However, pirfenidone exhibited cytotoxicity (77.57% survival rate), while compounds **1**, **4**, and **7** showed low cytotoxicity against the HFL1 cell line. Particularly, exophilone (**1**) showed moderate collagen deposition inhibition effect (60.44% inhibition rate) and low toxicity in HFL1 cells (98.14% survival rate) at a concentration of 10 μM. A molecular docking study suggests that exophilone (**1**) binds to both TGF-β1 and its receptor through hydrogen bonding interactions. Thus, exophilone (**1**) was identified as a promising anti-pulmonary fibrosis agent. It has the potential to be developed as a drug candidate for pulmonary fibrosis.

## 1. Introduction

Deep-sea is one of the extreme ecological environments on earth, with high salinity, high pressure, low temperature, low oxygen concentration, darkness, and other characteristics [1]. Therefore, organisms, including microbes, that live in deep-sea are normally equipped with certain physical and biochemical traits that help them survive that extreme environment [2]. In addition, many of them have the ability to produce specialized metabolites which are different from those produced by terrestrial organisms. Recent studies have shown that fungi from extreme environments have great potential as a source of clinically important compounds [1,3].

Tetrahydro carbazole derivatives have been isolated from microorganisms of terrestrial and marine origin and exhibit a variety of activities, including anti-*Candida albicans* activity [4], anti-*Bacillus subtilis* activity, and anti-*Micrococcus luteus* activity [5], etc. In particular, sorazolons D2, E, and E2 from *Sorangium cellulosum* strain Soce375 exhibited anti-fibrosis activity [5]. Pulmonary fibrosis (PF) is a lung disease in which scarring of the lungs increases over time [6]. The progression of PF is related to environmental pollution, certain drug use, connective tissue diseases, infections (including COVID-19 and the related SARS virus), and/or interstitial lung disease [7]. To date, only two drugs, nintedanib and pirfenidone, have been approved by the FDA for the treatment of idiopathic pulmonary fibrosis (IPF). Nintedanib can significantly slow disease progression compared to placebo in IPF patients [8,9]. However, its clinical applications are somewhat limited due to poor oral bioavailability, metabolic instability, and off-target side effects [10]. Clinical trials have shown that pirfenidone alleviates the decline in lung function in patients with IPF, but 24.3% of patients stopped pirfenidone treatment due to adverse drug reactions in Japan [11]. Although lung transplantation is considered the most effective treatment for PF, it is limited by the lack of suitable donor organs [12]. Therefore, there is still an urgent need to identify and discover new agents to treat PF. PF is characterized by excessive collagen deposition in the lung; therefore, an in vitro cell screening assay that is based on deposition of collagen in cells has been established [13].

**Figure 1:**
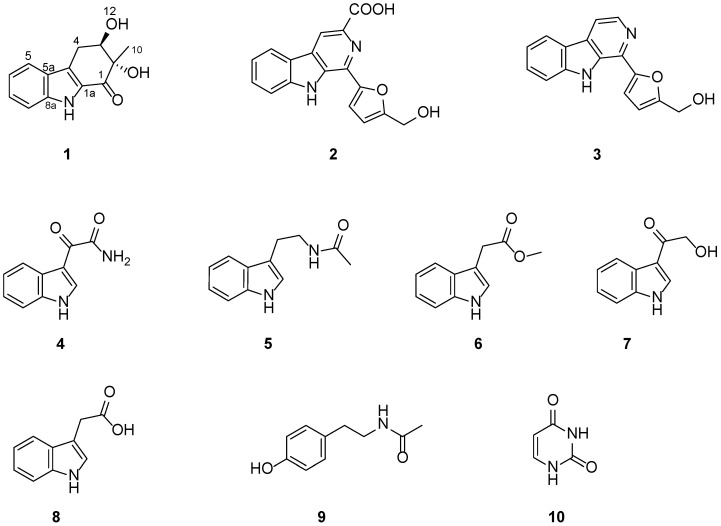
Chemical structures of compounds **1**–**10**.

As part of an effort to discover anti-PF compounds from extremophilic fungi, we investigated the metabolites of the fungus *Exophiala oligosperma* MCCC 3A01264, a “black-yeast” isolated from seawater collected at a depth of 3300 m in the northern basin of the South China Sea. While *E. oligosperma* has been reported to cause infections in humans, particularly in immunocompromised patients [3], little is known about its secondary metabolism or production of natural products. In this study, we focused our effort on bioactive compounds that have potential to be developed as drugs for the treatment of pulmonary fibrosis (PF). Here, we report the isolation, structure characterization, and collagen accumulation inhibitory activity of a new compound, exophilone (**1**), together with eleven known compounds (**2**–**10**) from *E. oligosperma* (Figure 1).

## 2. Results and Discussion

### 2.1. Structural Elucidation

**Figure 2:**
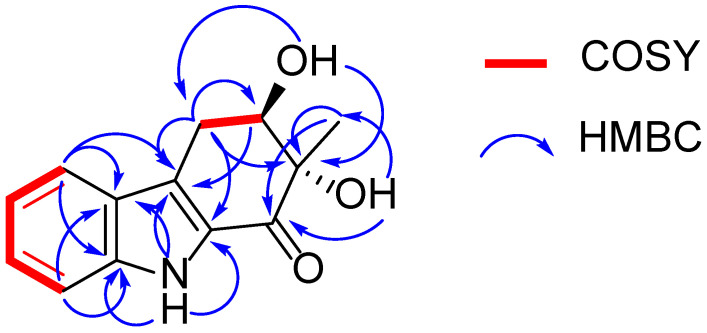
^1^H-^1^H COSY and key HMBC correlations of compound **1**.

Exophilone (**1**) was isolated as a pale yellow solid, and its molecular formula was established to be C_13_H_13_NO_3_ by HRESIMS (*m/z* 232.0967 [M + H]^+^, calcd. 232.0968) (Supplementary Figure S1), suggesting eight degrees of unsaturation. Analyses of its ^1^H, ^13^C, and HMQC NMR spectra (Table 1 and Supplementary Figures S2–S5) revealed the presence of one carbonyl (C-1), two aromatic quaternary (C-4a, C-5a), two tertiary amines (C-8a, C-1a), and four aromatic methines (C-5 to C-8), as well as one tertiary alcohol (C-2), one secondary alcohol (C-3), one methylene (C-4), and one methyl (C-10). The carbonyl and four double bonds accounted for five out of the eight degrees of unsaturation required by the molecular formula, and the remaining three suggested a structure with three rings in **1**. Based on the COSY correlations between H-5 (δ_H_ 7.66, d, *J* = 7.8 Hz) and H-6 (δ_H_ 7.08, ddd, *J* = 7.8, 7.2, 1.2 Hz), between H-6 and H-7 (δ_H_ 7.30, ddd, *J* = 7.8, 7.2, 1.2 Hz), between H-7 and H-8 (δ_H_ 7.38, d, *J* = 7.8 Hz), as well as HMBC correlations between H-5 and C-5a (δ_C_ 125.3), C-8a (δ_C_ 138.9), and C-4a (δ_C_ 123.6), between H-8 and C-5a (δ_C_ 125.3) and C-8a (δ_C_ 138.9), between H-9 (δ_H_ 11.59, s) and C-4a (δ_C_ 123.6), C-5a (δ_C_ 125.3), C-8a (δ_C_ 138.9), and C-1a (δ_C_ 129.5), the presence of 2,3-substituted indole moiety was confirmed (Figure 2 and Supplementary Figure S6–S7). The third ring was confirmed by C-1 (δ_C_ 192.4) to C-4 (δ_C_ 27.5), where C-1 attaches to C-1a and C-4 attaches to C-4a. This was supported by the HMBC correlation between C-4 methylene protons and C-4a, C-1a, C-2, and C-3, between the methylene protons and the carbonyl C-1, between the methyl protons CH_3_-10 (δ_H_ 1.24, s) and C-2, C-1, and between H-3 (δ_H_ 4.03, m) and C-4a suggested that C-1 is connected to C-2 and C-2 is connected to C-3 and C-10. Further, the HMBC spectrum allowed the assignment of the position of the OH groups (δ_H_ 5.25 and 5.16 ppm) to C-11 and C-12 from their correlations with C-1 and C-10 as well as C-2 and C-4, respectively (Figure 2 and Supplementary Figure S7). Hence, the structure of **1** was elucidated as shown (Figure 1).

**Figure 3:**
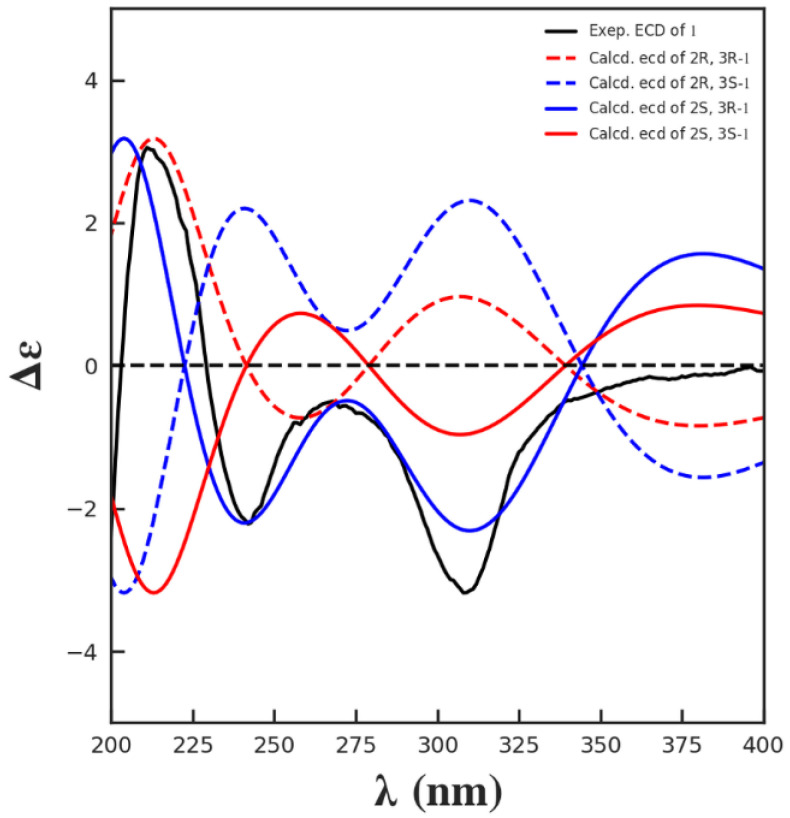
Comparisons of the experimental and calculated ECD spectra of **1**.

Since the NOESY spectrum of **1** (Supplementary Figure S8) did not provide enough information to determine its configuration, the absolute configuration of **1** was elucidated to be 2*S*, 3*R* by comparisons of the experimental and calculated electronic circular dichroism (ECD) spectra (Figure 3). Since the structure of **1** has not been reported previously, it is named exophilone (**1**).

The other eleven compounds were determined to be flazine (**2**) [14], perlolyrine (**3**) [15], (1H-indol-3-yl) oxoacetamide (**4**) [16], N-acetyl tryptamine (**5**) [17], indole-3-methylethanoate (**6**) [18], 3-(hydroxyacetyl)-indole (**7**) [19], indole-3-acetic acid (**8**) [20], N-acetyl-tyramine (**9**) [21], and uracil (**10**) [22] by comparing their NMR data (Supplementary Figures S9–S26) with those reported in the literature.

### 2.2. Effect of Compounds ***1**–**10*** on HFL1 Cell Viability

**Figure 4:**
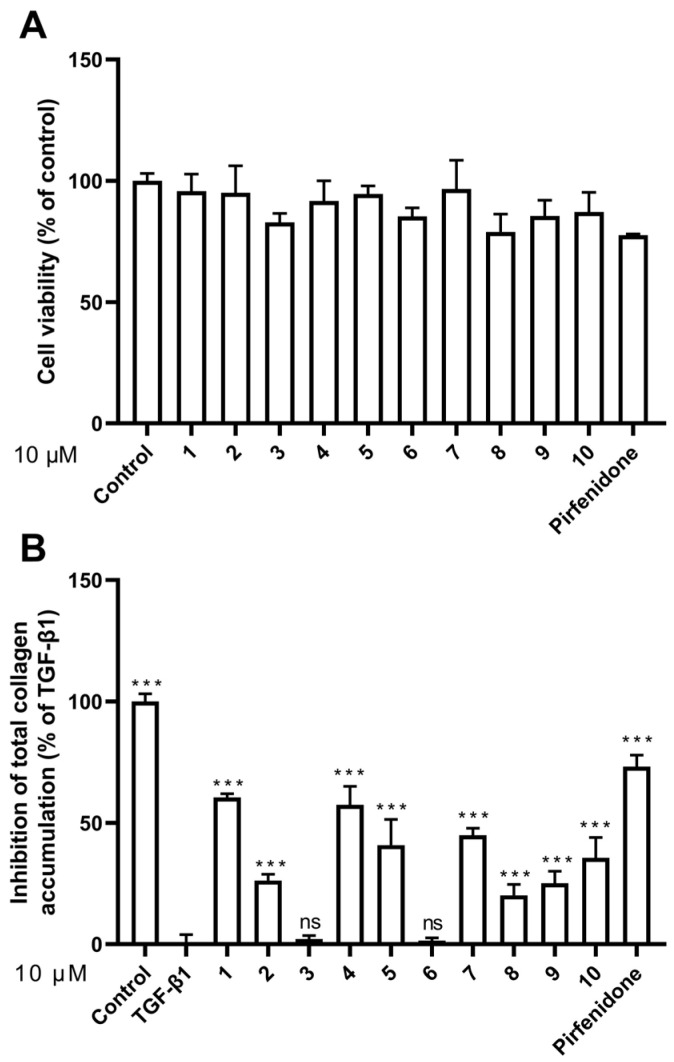
Effects of compounds **1–10** on cell viability and collagen accumulation. (**A**) Cell viability was calculated by CCK8 assay at a concentration of 10 μM; (**B**) Inhibitory activity against TGF-β1-induced total collagen accumulation in HFL1 cells at a concentration of 10 μM. The results are the means ± SD of at least three independent experiments. *** *p* < 0.001 compared with the TGF-β1 group. ns: no statistical difference.

To assess the cytotoxicity of compounds **1**–**10**, we performed cell viability assay with the HFL1 cell line. The cells were treated with compounds **1**–**10** as well as with pirfenidone as a positive control for 48 h, and the cell viability was measured and compared with the untreated control group (control) (Figure 4A and Table 2). Among the compounds tested, compound **8** and pirfenidone are somewhat toxic to HFL1 cells at 10 μM, with cell survival rates of 78.49% and 77.57%, respectively. On the other hand, compounds **1**, **2**–**7**, **9**, and **10** did not significantly affect cell viability at the same concentration. Particularly, compounds **1**, **4**, and **7** had no cytotoxicity at 10 μM, with the cell survival rates of above 98%.

### 2.3. Effect of Compounds ***1**–**10*** on HFL1 Cell Collagen Accumulation

**Figure 5:**
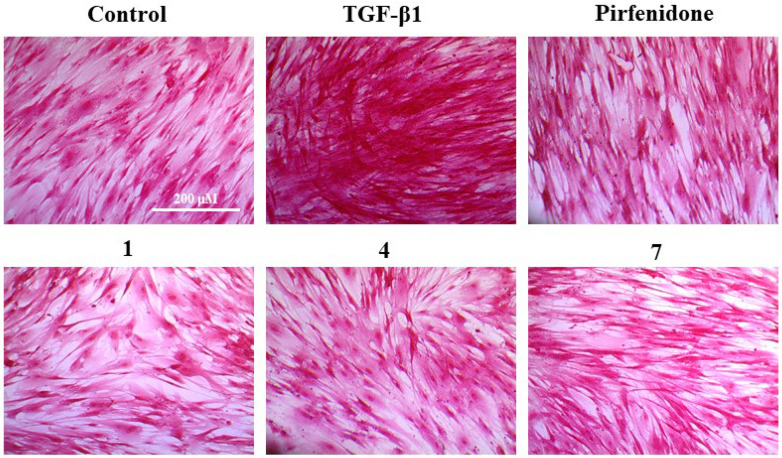
Picro-Sirius Red (PSR) staining for the total collagen accumulation induced by TGF-β1 in HFL1 cells. The representative images are the cells induced by TGF-β1 and treated with 10 μM of compounds **1**, **4**, **7**, pirfenidone, and the control group (untreated normal cells). Scale bar: 200 μM.

To evaluate the compounds’ inhibitory activity on TGF-β1-induced total collagen accumulation, the Sirius red dye staining, which has been accepted to be an effective and convenient method for the anti-fibrotic screening model in vitro [13,23], was used. Among the compounds tested, compounds **1**, **4**, and **7** showed good inhibition of collagen accumulation (60.44%, 57.37%, and 44.96%) in HFL1 cells (Table 2 and Figure 4B). While they are somewhat less active than pirfenidone, their toxicity profiles are less than pirfenidone (77.57% survival rate) toward HFL1 cells. More significantly, exophilone (**1**) showed a respectable collagen deposition inhibition effect (60.44% inhibition rate) and low toxicity toward HFL1 cells (98.14% survival rate) at a concentration of 10 μM. The cells were observed with Picro-Sirius Red staining and visualized (Figure 5).

### 2.4. Molecular Docking Study

**Figure 6:**
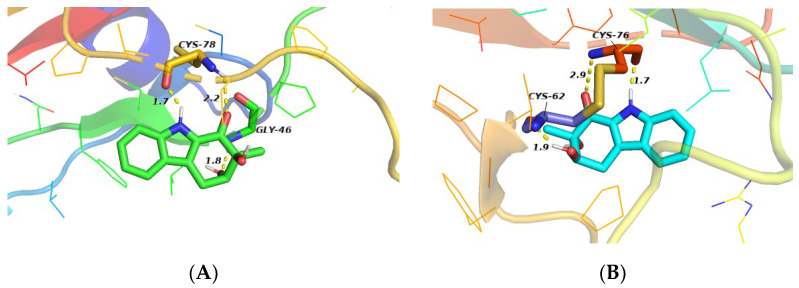
Molecular docking studies of compound **1**. (**A**) Docking mode of compound **1** to 1KLS; (**B**) Docking mode of compound **1** to 3KFD (Yellow dotted lines represent hydrogen bonds, and numbers represent bond distances).

The inhibitory effect of compound **1** on TGF-β1-induced total collagen accumulation in HFL1 cells might be due to its competitive binding with TGF-β1 (PDBID: 1KLS) or with its receptors (PDBID: 3KFD). In order to investigate the binding mode of compound **1,** molecular docking experiments were performed using Autodock software 1.56 [24]. The results are shown in Figure 6.

The docking study showed three hydrogen bonds between compound **1** and the active site residues of TGF-β1 (1KLS) (Figure 6A); a strong hydrogen bond (distance: 1.7 Å) between the indole nitrogen atom and the Cys-78 residue of TGF-β1, and two hydrogen bonds (distance: 2.2, 1.8 Å) between the two hydroxyl groups and Cys-78 and Gly-46, respectively. The data suggest that compound **1** may inhibit TGF-β1-induced total collagen accumulation in HFL1 cells by directly binding to TGF-β1. However, the study also showed three hydrogen bonds between compound **1** and the TGF-β1 receptor (3KFD) (Figure 6B); one hydrogen bond (distance: 1.7 Å) between the indole nitrogen atom and Cys-76, a hydrogen bond (distance: 2.9 Å) between the C-1 ketone and Cys-76, and another hydrogen bond between the C-3 hydroxyl group and Cys-62 (distance: 1.9 Å). The results suggest that compound **1** may bind to the active site of the TGF-β1 as well as to its receptor by hydrogen bonding interactions. These may preliminarily explain why compound **1** inhibits the accumulation of collagen induced by TGF-β1 similar to pirfenidone in HFL1 cells. The interactions of compound **1** with TGF-β1 and its receptor will be a subject of our future investigations.

## 3. Discussion

Exophilone (**1**) is a tetrahydro carbazole derivative that is structurally very similar to Sorazolon A [5], which was previously found in soil-derived *Sorangium cellulosum* strain soce375 and thus presumably has a similar biosynthetic pathway. The main differences between exophilone (**1**) and Sorazolon A are the carbonyl C-1 and the secondary alcohol C-3 replacing the tertiary alcohol and carbonyl. Natural tetrahydro carbazole derivatives, including 3-hydroxy-1,2-dimethyl-1,2,3,9-tetrahydrocarbazol-4-one isolated from *Streptomyces ehimensis* strain JB201 [4] and carbazomycin dimers and 3-hydroxy-1,2-dimethyl-2,3-dihydro-1*H*-carbazol-4-one isolated from *Streptomyces* sp. BCC26924 [25], showed antifungal activity and antituberculosis activity. Furthermore, synthetic tetrahydro carbazole protects DNA against oxidative stress [26]. Natural products are a rich source of lead molecules for anti-fibrosis drug discovery. Current pulmonary fibrosis treatment drugs (e.g., colchicine, cyclophosphamide, cyclosporine A, pirfenidone, and nitinol) have therapeutic effects but also significant side effects. Therefore, it is crucial to screen drugs with progressive therapeutic effects to treat pulmonary fibrosis [27].

In this study, a new compound, exophilone (**1**), together with nine known compounds (**2**–**10**), were isolated from a deep-sea-derived fungus, *Exophiala oligosperma*. Among them, exophilone (**1**) showed the best anti-pulmonary fibrosis activity, with low toxicity in HFL1 cells (98.14% survival rate) at a concentration of 10 μM. Exophilone (**1**) has the potential of anti-pulmonary fibrosis and may bind to both TGF-β1 and its receptor through hydrogen bonding interactions.

## 4. Materials and Methods

### 4.1. General Procedures

The PerkinElmer Spectrum Two spectrometer (PerkinElmer, Waltham, MA, USA) was used for IR spectra measurement. ECD spectra were measured on a Chirascan circular dichroism spectrometer (Applied Photophysics Ltd., Leatherhead, UK). UV spectra were obtained on a Shimadzu UV-vis-NIR spectrophotometer (Shimadzu Corporation, Nakagyo-ku, Kyoto, Japan). 1D and 2D NMR spectra were recorded in CDCl_3_ or DMSO-*d*6 on Bruker Avance II 400, Bruker Avance IIIT 500HD, Bruker Avance IIIT 600AV spectrometers (Bruker Bio Spin AG, Industriestrasse 26, Fällanden, Switzerland). The chemical shifts are relative to the residual solvent signals (CDCl_3_: δ_H_ 7.260 and δ_C_ 77.160; DMSO-*d*6: δ_H_ 2.500, δ_C_ 39.520). The high-resolution ESI-MS spectra were obtained on a Thermo Fisher LTQ Orbitrap Elite High-Resolution liquid chromatography-mass spectrometer (Thermo Fisher Scientific Inc., Waltham, MA, USA). Preparative HPLC was performed using a Shimadzu LC-15C HPLC pump (Shimadzu Corporation, Nakagyo-ku, Kyoto, Japan) supplied with an SPD-15C dual λ absorbance detector (Shimadzu Corporation, Nakagyo-ku, Kyoto, Japan), and a Shim-pack PRC-ODS HPLC column I (250 × 20 mm i.d., 5 µm, Shimadzu Corporation, Nakagyo-ku, Kyoto, Japan). Silica gel (SiO_2_, 200–300 mesh, Qingdao Puke parting Materials Co., Ltd. Qingdao, China) and Sephadex LH-20 (green herbs, Beijing, China) were used for column chromatography.

### 4.2. Fungal Strain and Culture Method

The marine fugus *Exophiala oligosperma* MCCC 3A01264 was obtained from Marine Culture Collection of China (MCCC). It was originally isolated from seawater collected at a depth of 3300 m in northern basin of the South China Sea. A voucher specimen was stored in the School of Pharmaceutical Sciences, Sun Yat-sen University, Guangzhou, P.R. China. Analysis of the internal transcribed spacer (ITS) rDNA by BLAST database screening provided a 99.9% match to *Exophiala oligosperma*.

The fermentation medium contained glucose (15 g/L), peptone (10 g/L), yeast extract (2 g/L), L-tryptophan (2 g/L), L-phenylalanine (2 g/L), L-methionine (2 g/L), L-threonine (2 g/L), sea salt (25 g/L), and H_2_O (1 L), and was adjusted to pH 7.5. Fungal mycelia were cut uniformly and transferred aseptically to 1 L Erlenmeyer flasks with each containing 600 mL liquid medium sterilized at 120 °C for 30 min. The flasks were incubated at 28 °C for 60 days.

### 4.3. Extraction and Isolation

Two hundred liters of culture broth were filtered through the cheesecloth. The culture broth was extracted three times using EtOAc and then concentrated under reduced pressure to afford an EtOAc extract (31.68 g).

The EtOAc extract was chromatographed on a silica gel column (diameter: 80 mm, length: 610 mm, silica gel: 400 g) with a gradient of petroleum ether–EtOAc (10:0–0:10, *v*/*v*) followed by EtOAc–MeOH (10:0–0:10, *v*/*v*) to afford 10 fractions (coded Fr.1–Fr.10). Compound **8** (320 mg) was crystallized from Fr.6 severally. Fr.7 (1.2 g) was subjected to a silica gel column (7 g) eluted with petroleum ether-EtOAc (100:0−0:100) (total volume 2 L) with increasing polarity to obtain ten subfractions (Fr.7-1−Fr.7-10) after pooling the similar fractions as monitored by TLC (petroleum ether−EtOAc = 4:1). Compound **6** (4.2 mg) was obtained from Fr.7-4 directly. Fraction 8 (143 mg) was chromatographed on Sephadex LH-20 (10 g) and eluted with MeOH (total volume 500 mL) to give five subfractions (Fr.8-1−Fr.8-5). Fr.8-4 was further fractionated by preparative HPLC (MeOH–H_2_O, 55:45, *v*/*v*, column I) to yield compound **1** (2.1 mg, TR = 22 min), compound **7** (3.0 mg, TR = 16.5 min), and compound **4** (4.2 mg, TR = 25 min). Compound **2** was filtered from Fr.10 directly. The rest of Fr.10 was separated by silica gel column using a step gradient elution with petroleum ether–EtOAc (10:0–0:10) to get 5 subfractions (Fr.10-1–Fr.10-5). Compound **5** (RT = 40.2 min, 3 mg), compound **3** (RT = 23 min, 8 mg), and compound **9** (RT = 36 min, 6 mg) were obtained from Fr.10-2 by preparative HPLC (MeOH–H_2_O, 80:20, *v*/*v*, column I). Compound **10** (RT = 15 min, 2 mg) was isolated by preparative HPLC (MeOH–H_2_O, 80:20, *v*/*v*, column I).

Exophilone (**1**). UV (MeOH) λmax (logε) 307 (1.20), 233 (1.29), 206 (2.02). ECD (0.3 mM, MeOH) λmax (∆ε) 211 (+3.05) nm. IR υ_max_ 3287, 2922, 2852, 1716, 1651, 1456, 1374, 1330, 1237, 1152, 1097, 1070, 1044, 998, 920, 744, 554 cm^−1^. ^1^H and ^13^C NMR data, shown in Table 1; HR-ESI-MS *m/z* 232.0967 [M + H]^+^ (calcd. for C_13_H_13_NO_3_, 232.0968).

### 4.4. Cell Culture and Cytotoxicity Assay

The human fetal lung fibroblasts (HFL1) were purchased from Procell Life Science & Technology Co., Ltd. (Cat. No.: CL-0106 Wuhan, China). Cells were cultured in Ham’s F-12K medium (PM150910, Procell Life Science & Technology, Wuhan, China) supplemented with 10% fetal bovine serum (FBS) (#10270-106, GIBCO, Invitrogen, Carlsbad, CA, USA) and 1% penicillin-streptomycin in an incubator at 37 °C with 5% CO_2_. The cell viability was assayed using the Cell Counting Kit-8 (CCK8) according to the manufacturer’s protocol. The cells were treated with 10 μM compounds **1**-**10**, or pirfenidone (TargetMol, Wellesley Hills, MA, USA) for 48 h. The absorbance of the solution was then measured at 450 nm using a microplate reader (Thermo Fisher, Waltham, MA, USA). Survival rate = (Administration A value − Blank A value)/(Control A value − Blank A value) × 100%. All assays were repeated in triplicate.

### 4.5. Collagen Accumulation Inhibition In Vitro

The anti-fibrosis activities of the compounds were tested in HFL1 cells. The cells were treated with medium containing TGF-β1 (5 ng/mL) and 10 μM compounds **1**-**10**, and pirfenidone for 48 h. Subsequently, the supernatant was removed, and 4% paraformaldehyde was added to fix for 30 min at room temperature. Next, the cells were washed with PBS twice and then were added the 0.1% Sirius red dye with saturated picric acid. After 4 h of staining protected from light, the collagenous fiber was dyed red. Then, the cells were washed three times with 0.1% acetic acid and visualized under a cell imaging system (EVOS FL Auto, Life Technologies, Carlsbad, CA, USA). For the quantitative determinations of the accumulated collagen, the stained cells were destained with 0.1 M NaOH (100 μL/well) for 10 min. Then, the absorbance was measured at 540 nm with a spectrophotometer. Total collagen accumulation inhibition = 1 − (Administration A value − control A value)/(model A value − control A value) × 100%. All assays were repeated in triplicate.

### 4.6. Molecular Docking

Protein structure was obtained from the Protein Data Bank (https://www.pdbus.org/, accessed on 29 May 2022). The X-ray crystal structure of TGF-β1 (PDB ID: 1LKS) and its receptor (PDB ID: 3KFD) were chosen for the molecular docking analysis in this study. Compound **1** was prepared with Avogadro 1.1.1, with a 5000 steps Steepest Descent as well as 1000 steps Conjugate Gradients geometry optimization using MMFF94 force field. Docking experiments were performed using AutoDock 1.56 Vina and Pymol 2.4.

### 4.7. Statistical Analysis

The data are represented as the mean ± SD. Statistical analysis was performed using the GraphPad Prism 8.0 software (San Diego, CA, USA). The significant differences between groups were statistically analyzed using the one-way analysis of variance (ANOVA) followed by a post hoc test (LSD). All differences were considered statistically significant at *p* < 0.05.

## 5. Conclusions

A new tetrahydrocarbazol-1-one analogue, exophilone (**1**), together with nine known compounds (**2**–**10**), were isolated from a deep-see-derived fungus *Exophiala oligosperma*. Among all compounds, exophilone (**1**) showed the most significant inhibition of collagen accumulation with low toxicity in HFL1 cells. Further molecular docking experiments showed that exophilone (**1**) may act through hydrogen bonding to the stimulation site of TGF-β1 and its receptor. Given the limitations of the available anti-pulmonary fibrosis drugs, exophilone (**1**) and its analogs could be developed as candidates for the treatment of pulmonary fibrosis.

## Figures and Tables

**Table 1:** ^1^H (400 Hz) and ^13^C (100 Hz) NMR data for compound **1** in DMSO-*d*6.

Position	δ_C_, Type	δ_H_, Mult. (*J* in Hz)
1	192.4, CO	
2	77.3, C	
3	74.2, CH	4.03, m
4	27.5, CH_2_	2.76, m 3.28, m
4a	123.6, C	
5a	125.3, C	
5	121.2, CH	7.66, d (7.8)
6	119.7, CH	7.08, ddd (7.8, 7.2, 1.2)
7	126.2, CH	7.30, ddd (7.8, 7.2, 1.2)
8	112.8, CH	7.38, d (7.8)
8a	138.9, C	
9	NH	11.59, brs
1a	129.5, C	
10	18.6, CH_3_	1.24, s
11	OH	5.25, brs
12	OH	5.16, d (3.6)

**Table 2:** Collagen accumulation inhibition rate (IR) and cell survival rate (SR) of **1**-**10**.

Compound	Inhibition (%)	Survival Rate (%)
**1**	60.44 ± 1.54	98.14 ± 6.20
**2**	26.28 ± 2.53	98.28 ± 11.15
**3**	2.19 ± 1.34	81.69 ± 3.36
**4**	57.37 ± 7.65	91.56 ± 10.17
**5**	40.88 ± 10.52	96.11 ± 1.80
**6**	1.46 ± 1.16	87.01 ± 1.13
**7**	44.96 ± 2.82	99.25 ± 12.96
**8**	20.15 ± 4.49	78.49 ± 8.92
**9**	25.11 ± 4.97	84.93 ± 7.86
**10**	35.62 ± 8.39	86.96 ± 9.81
pirfenidone	73.14 ± 4.72	77.57 ± 0.52

Inhibitory effect against TGF-β1 induced total collagen accumulation in HFL1 cells at a concentration of 10 μM. Cell survival rate is calculated by CCK8 assay. The results are the mean ± SD of at least three independent experiments.

## Data Availability

All relevant data are available from the corresponding author upon reasonable request.

## Supplementary Materials

The following are available online at https://www.mdpi.com/article/10.3390/md20070448/s1. Figures S1–S26: The HR-(+)ESI-MS and NMR spectra of compounds **1**–**10**; Figures S27–S30: NMR spectra of Compounds **11** and **12**.
