# Supplementary material for: Exophilone, a Tetrahydrocarbazol-1-one Analogue with Anti-Pulmonary Fibrosis Activity from the Deep-Sea Fungus Exophiala oligosperma MCCC 3A01264
Source: Mar Drugs. 2022 Jul 9;20(7):448. doi: 10.3390/md20070448 (PMC9317524; doi:10.3390/md20070448)
Supplement: Supplementary file 1 [file marinedrugs-20-00448-s001.zip › marinedrugs-1788817-supplementary.pdf]

Supplementary Material

Exopzolons with Anti-Pulmonary Fibrosis Activity from the Deep-Sea Fungus *Exophiala oligosperma* MCCC 3A01264

Ming-Jun Hong [1,†], Meng-Jiao Hao [1,†], Guang-Yu Zhang [1], Hou-Jin Li [2], Zong-Ze Shao [3], Xiu-Pian Liu [3], Wen-Zhe Ma [4], Jun Xu [1], Taifo Mahmud [5] and Wen-Jian Lan [1,*]

[1] School of Pharmaceutical Sciences, Sun Yat-sen University, Guangzhou 510006, China; hongmj5@mail2.sysu.edu.cn (Ming-Jun Hong); haomj@mail2.sysu.edu.cn (Meng-Jiao Hao); zhanggy39@mail2.sysu.edu.cn (G.-Y.Z.); xujun9@mail.sysu.edu.cn (J.X.)
[2] School of Chemistry, Sun Yat-sen University, Guangzhou 510006, China; ceslhj@mail.sysu.edu.cn (H.-J.L.)
[3] Key Laboratory of Marine Biogenetic Resources, Third Institute of Oceanography, Ministry of Natural Resources, Xiamen 361005, China; shaozz@163.com (Z.-Z.S.); mccc5177@163.com (X.-P.L.)
[4] State Key Laboratory of Quality Research in Chinese Medicine, Macau University of Science and Technology, Taipa 519020, Macau, China; wzma@must.edu.mo (W.-Z.M.)
[5] Department of Pharmaceutical Sciences, Oregon State University, Corvallis, OR 97331, USA; taifo.mahmud@oregonstate.edu
* Correspondence: lanwj@mail.sysu.edu.cn; Tel.: +86-20-399-43-042
† These authors contributed equally to this work.

List of contents:

Figure S1. HR-ESI-MS spectrum of Exopzolons (**1**). 3
Figure S2. ^{1}H NMR spectrum of Exopzolons (**1**) in DMSO-*d6* (600 MHz). 4
Figure S3. ^{13}C NMR spectrum of Exopzolons (**1**) in DMSO-*d6* (600 MHz). 5
Figure S4. DEPT 135 spectrum of Exopzolons (**1**) in DMSO-*d6* (600 MHz). 6
Figure S5. HMQC spectrum of Exopzolons (**1**) in DMSO-*d6* (600 MHz). 7
Figure S6. ^{1}H-^{1}HCOSY spectrum of Exopzolons (**1**) in DMSO-*d6* (600 MHz). 8
Figure S7. HMBC spectrum of Exopzolons (**1**) in DMSO-*d6* (600 MHz). 9
Figure S8. NOEY spectrum of Exopzolons (**1**) in DMSO-*d6* (600 MHz). 10
Figure S9. ^{1}H NMR spectrum of indole-3-acetic (**2**) in Acetone-*d6* (400 MHz). 11
Figure S10. ^{13}C NMR spectrum of indole-3-acetic (**2**) in Acetone-*d6* (400 MHz). 12
Figure S11. ^{1}H NMR spectrum of methyl Indol-3-ylacetate (**3**) in Acetone-*d6* (400 MHz). 13
Figure S12. ^{13}C NMR spectrum of methyl Indol-3-ylacetate (**3**) in Acetone-*d6* (400 MHz). 14
Figure S13. ^{1}H NMR spectrum of bis-(2-ethylhexyl) phthalate (**4**) in Acetone-*d6* (400 MHz). 15
Figure S14. ^{13}C NMR spectrum of bis-(2-ethylhexyl) phthalate (**4**) in Acetone-*d6* (400 MHz). 16
Figure S15. ^{1}H NMR spectrum of 3-(hydroxyl-acetyl)-1H-indole (**5**) in DMSO-*d6* (500 MHz). 17
Figure S16. ^{13}C NMR spectrum of 3-(hydroxyl-acetyl)-1H-indole (**5**) in DMSO-*d6* (500 MHz). 18
Figure S17. ^{1}H NMR spectrum of 2-(1H-indol-3-yl)-2-oxoacetamide (**6**) in DMSO-*d6* (500 MHz). 19
Figure S18. ^{13}C NMR spectrum of 2-(1H-indol-3-yl)-2-oxoacetamide (**6**) in DMSO-*d6* (500 MHz). 20
Figure S19. ^{1}H NMR spectrum of flazine (**7**) in DMSO-*d6* (400 MHz). 21
Figure S20. ^{13}C NMR spectrum of flazine (**7**) in DMSO-*d6* (400 MHz). 22
Figure S21. ^{1}H NMR spectrum of N-acetyltryptamine (**8**) in $CDCl_3$ (500 MHz). 23
Figure S22. ^{13}C NMR spectrum of N-acetyltryptamine (**8**) in $CDCl_3$ (500 MHz). 24
Figure S23. ^{1}H NMR spectrum of perlolyrine (**9**) in Acetone-*d6* (400 MHz). 25

Figure S24. ^{13}C NMR spectrum of perlolyrine (**9**) in Acetone-*d*6 (400 MHz). 26
Figure S25. ^{1}H NMR spectrum of N-acetyltyramine (**10**) in MeOD (400 MHz). 27
Figure S26. ^{13}C NMR spectrum of N-acetyltyramine (**10**) in MeOD (400 MHz)........ 28
Figure S27. ^{1}H NMR spectrum of uracil (**11**) in DMSO (400 MHz)........ 29
Figure S28. ^{13}C NMR spectrum of uracil (**11**) in DMSO (400 MHz). 30
Figure S29. ^{1}H NMR spectrum of cinnamic acid (**12**) in $CDCl_3$ (400 MHz). 31
Figure S30. ^{13}C NMR spectrum of cinnamic acid (**12**) in $CDCl_3$ (400 MHz)........ 32

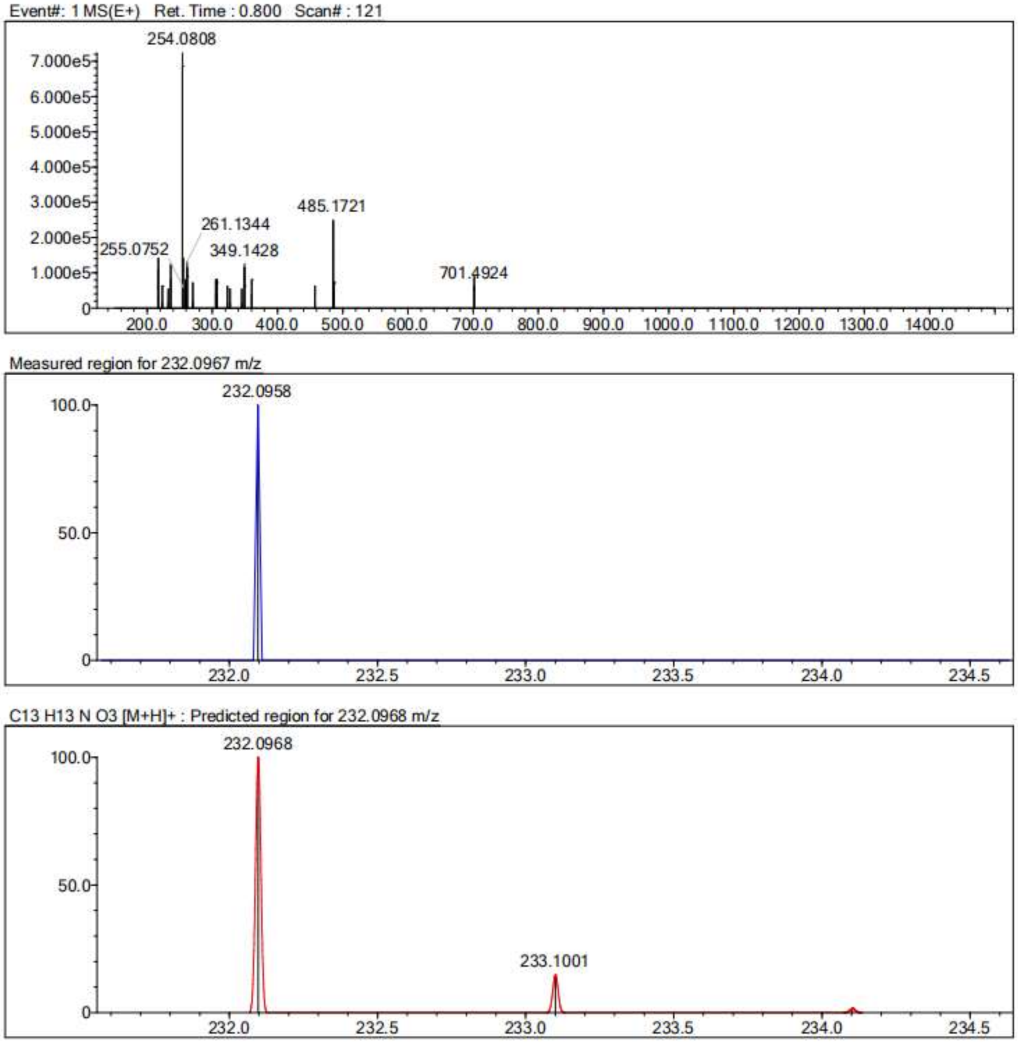

Rank	Score	Formula (M)	Ion	Meas. m/z	Pred. m/z	Df. (mDa)	Df. (ppm)	Iso	DBE
1	0.00	C13 H13 N O3	[M+H]+	232.0967	232.0968	-0.1	-0.43	0.00	8.0

Figure S1. HR-ESI-MS spectrum of Exopzolons (**1**).

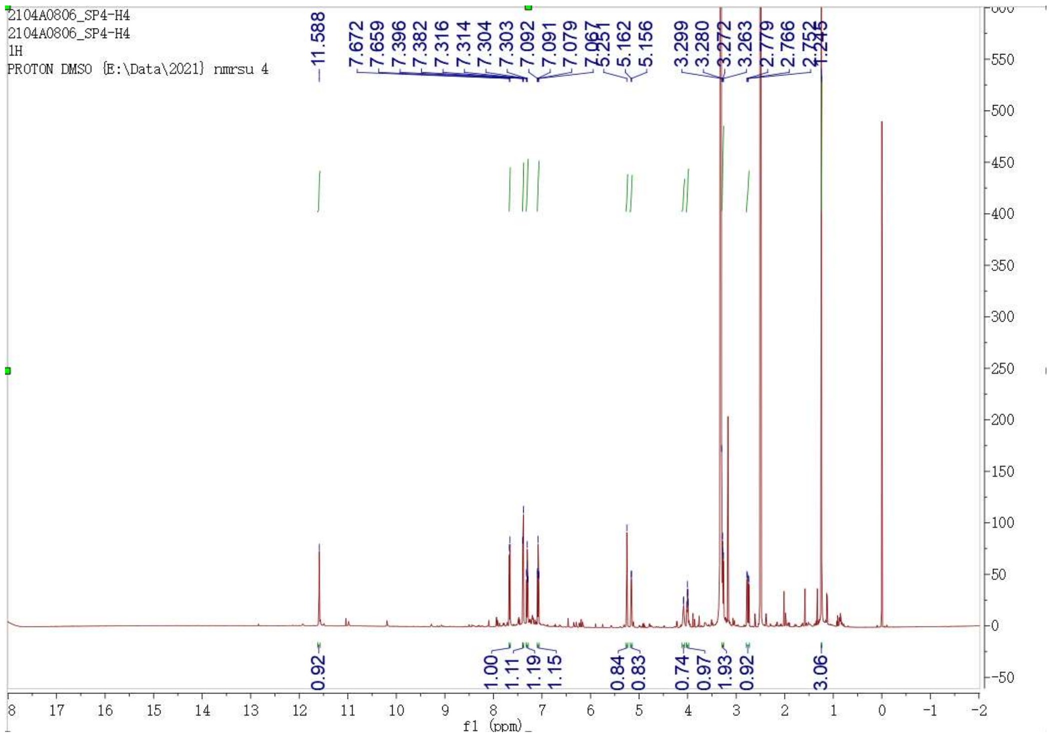

Figure S2. ^{1}H NMR spectrum of Exopzolons (**1**) in DMSO-*d*6 (600 MHz).

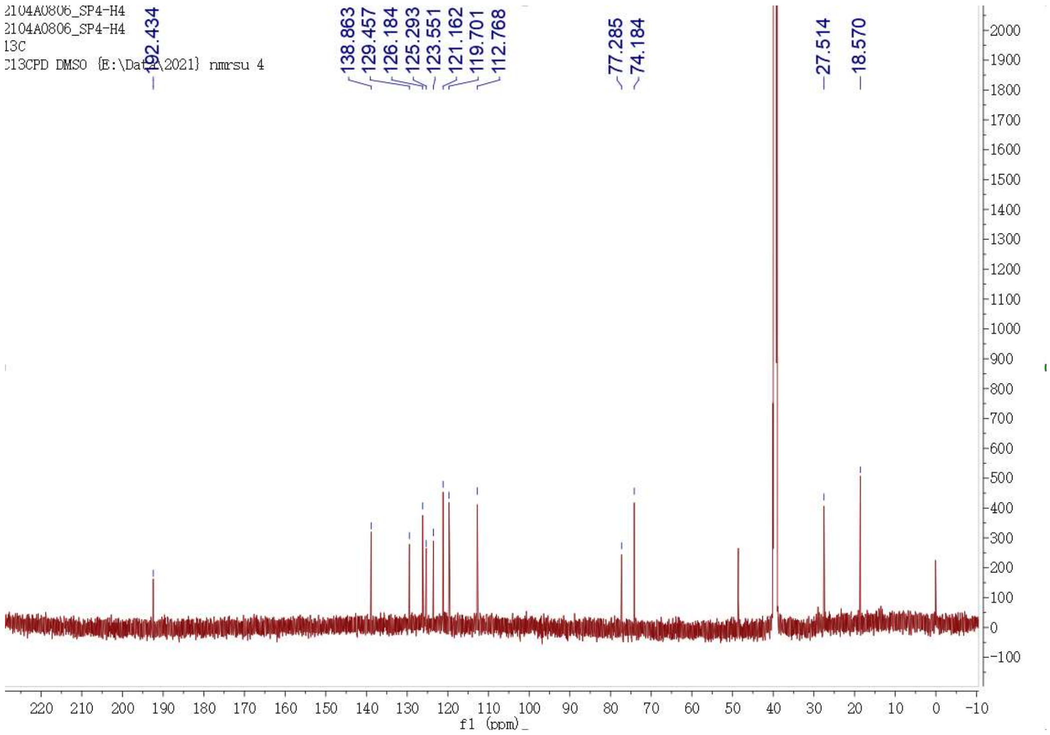

Figure S3. ^{13}C NMR spectrum of Exopzolons (**1**) in DMSO-*d*6 (600 MHz).

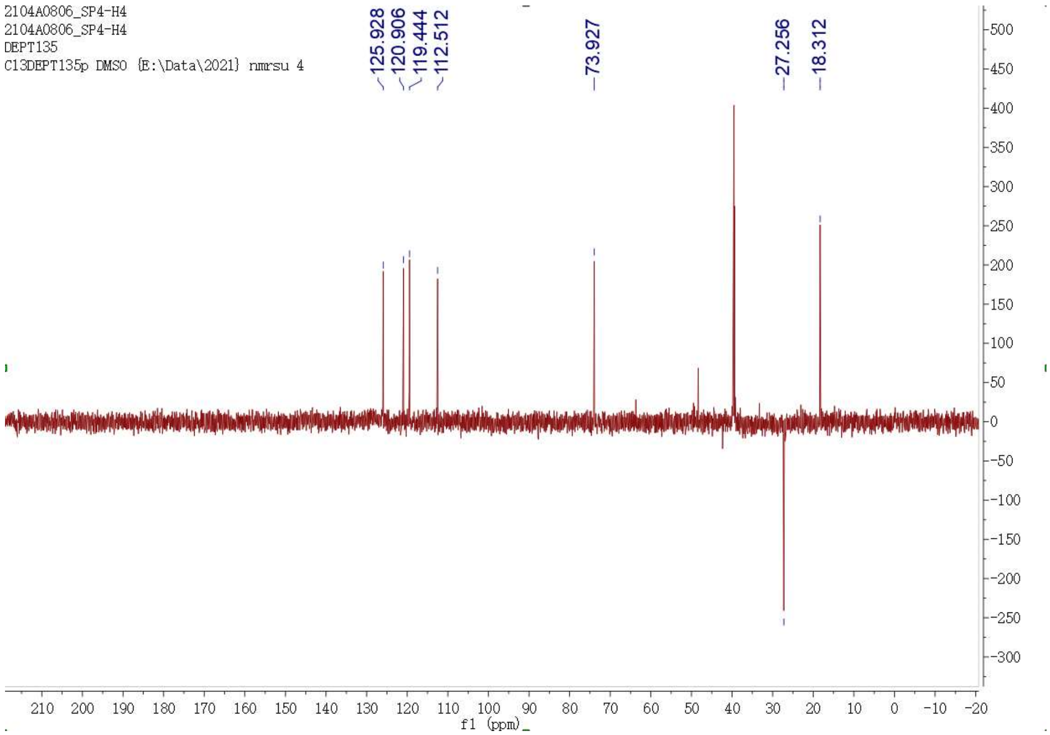

Figure S4. DEPT 135 spectrum of Exopzolons (**1**) in DMSO-*d*6 (600 MHz).

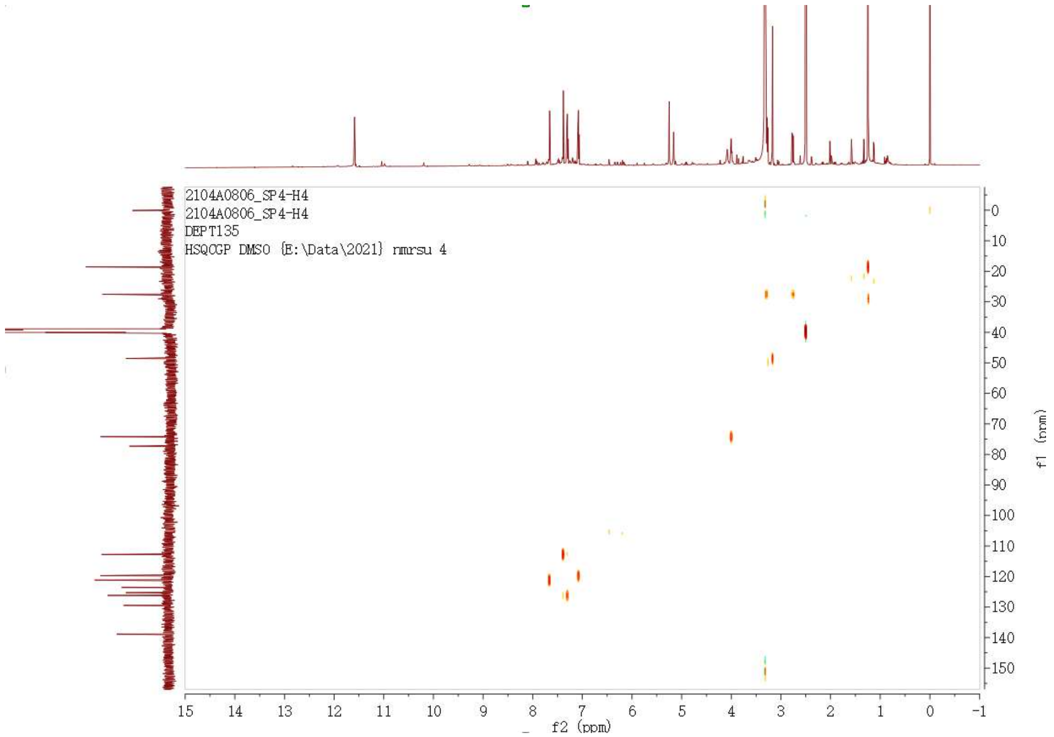

Figure S5. HMQC spectrum of Exopzolons (**1**) in DMSO-*d*6 (600 MHz).

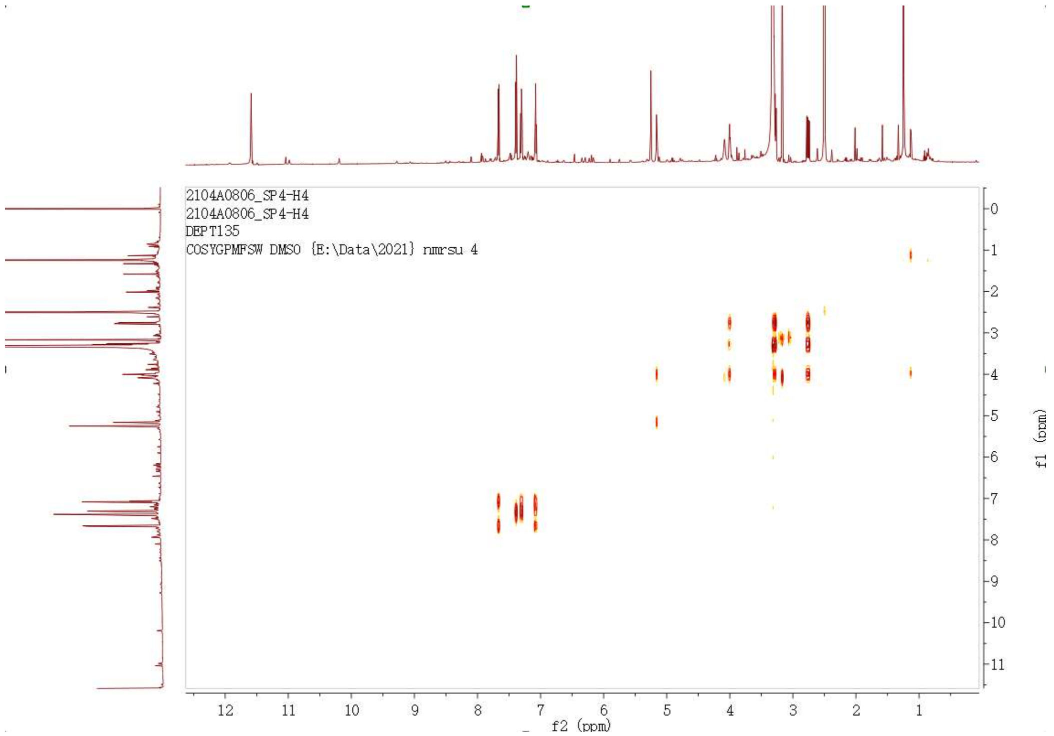

Figure S6. ^{1}H-^{1}HCOSY spectrum of Exopzolons (**1**) in DMSO-*d*6 (600 MHz).

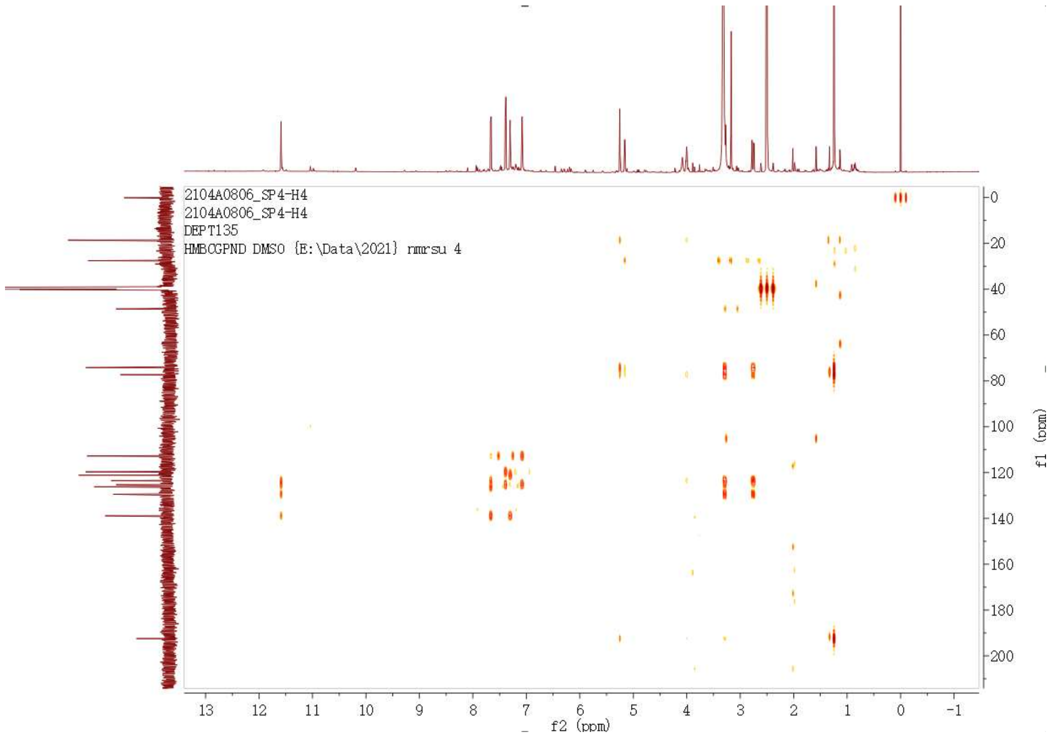

Figure S7. HMBC spectrum of Exopzolons (**1**) in DMSO-*d*6 (600 MHz).

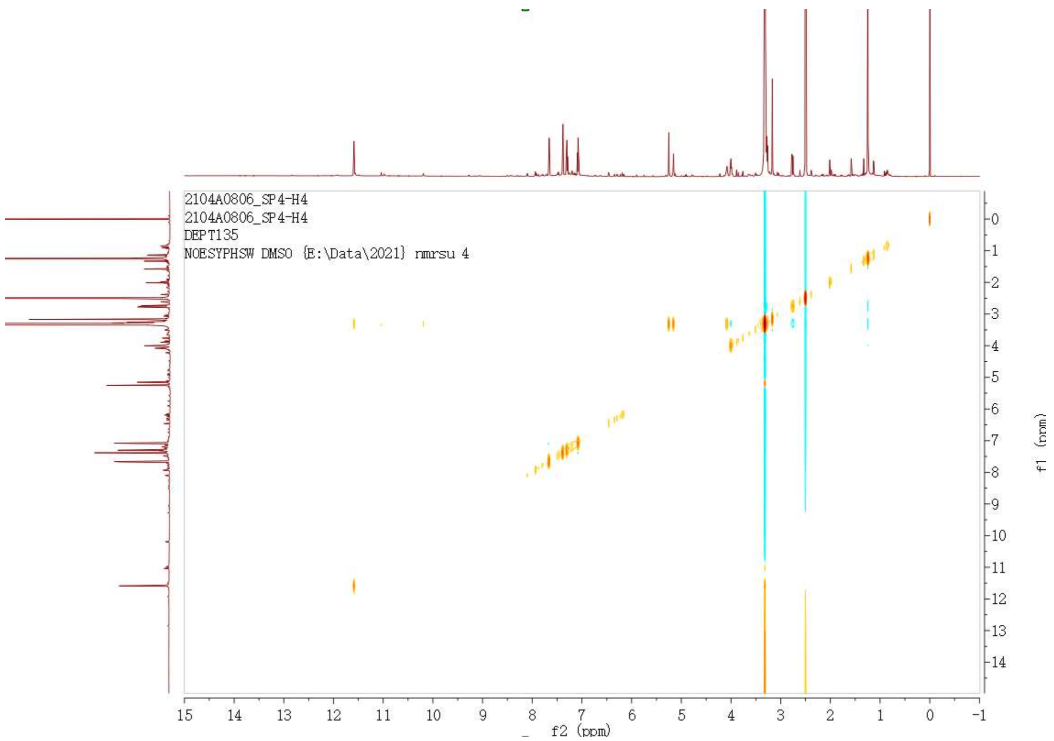

Figure S8. NOEY spectrum of Exopzolons (**1**) in DMSO-*d*6 (600 MHz).

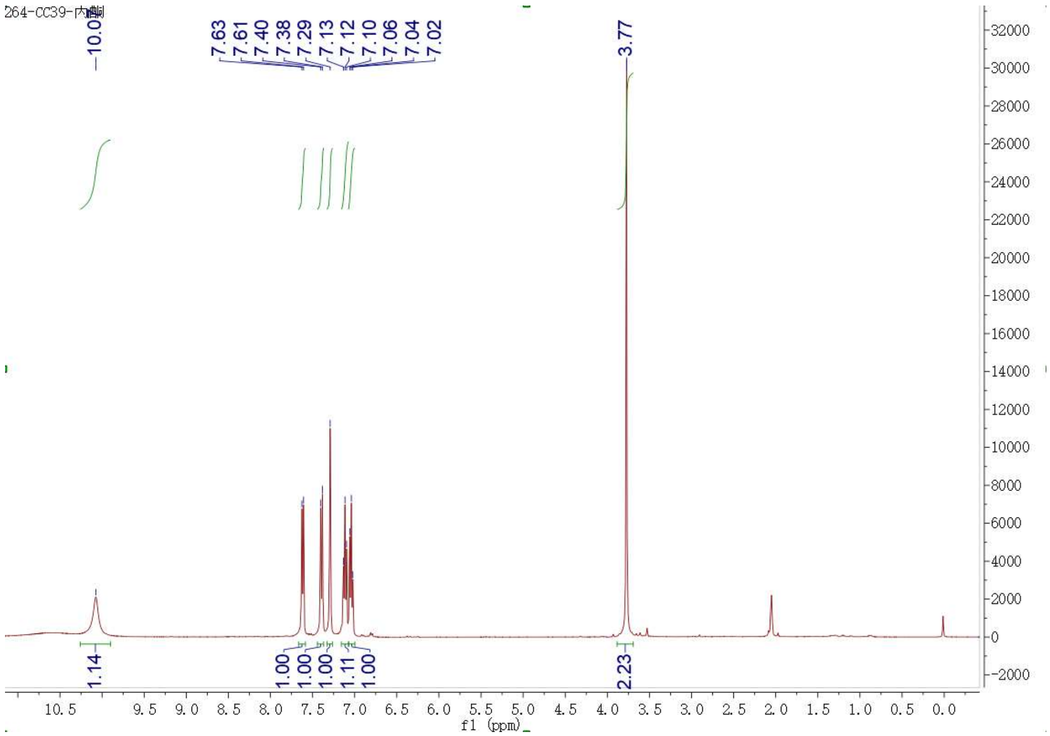

Figure S9. ^{1}H NMR spectrum of indole-3-acetic (**2**) in Acetone-*d*6 (400 MHz).

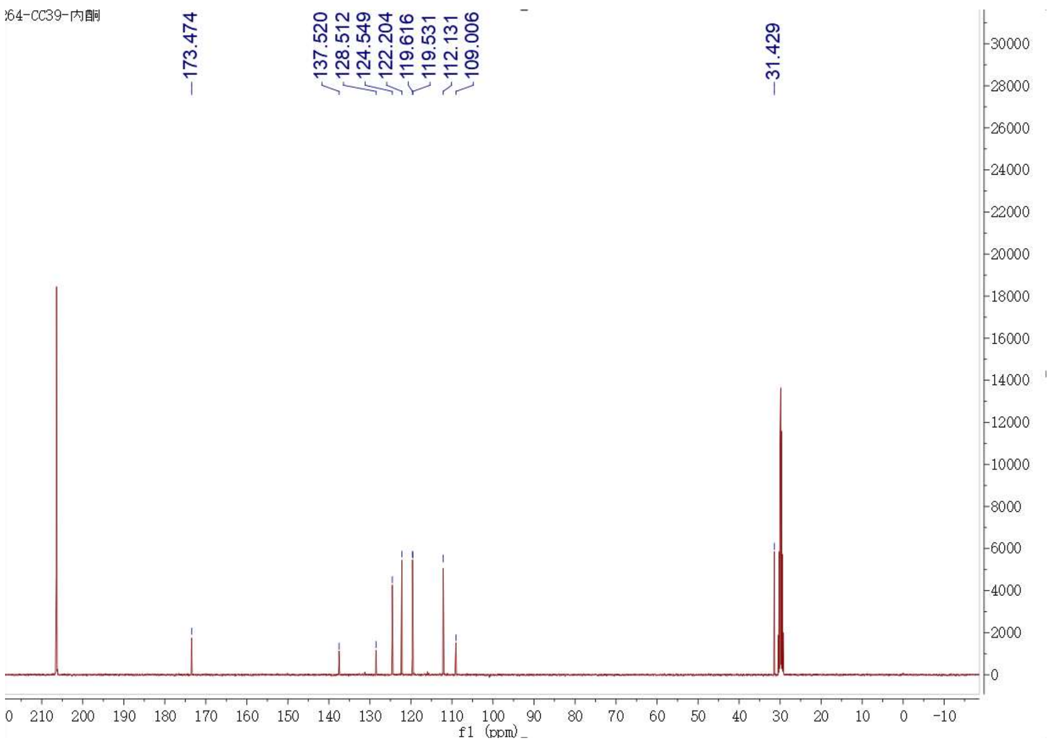

Figure S10. ^{13}C NMR spectrum of indole-3-acetic (**2**) in Acetone-*d*6 (400 MHz).

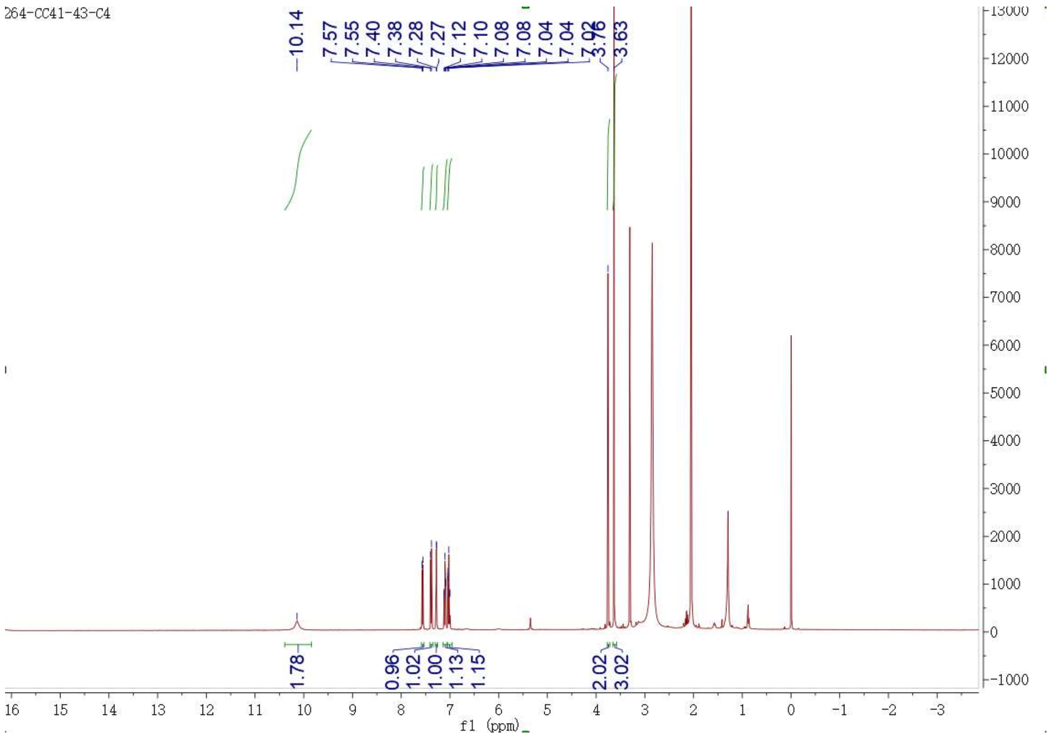

Figure S11. ^{1}H NMR spectrum of methyl Indol-3-ylacetate (**3**) in Acetone-*d*6 (400 MHz).

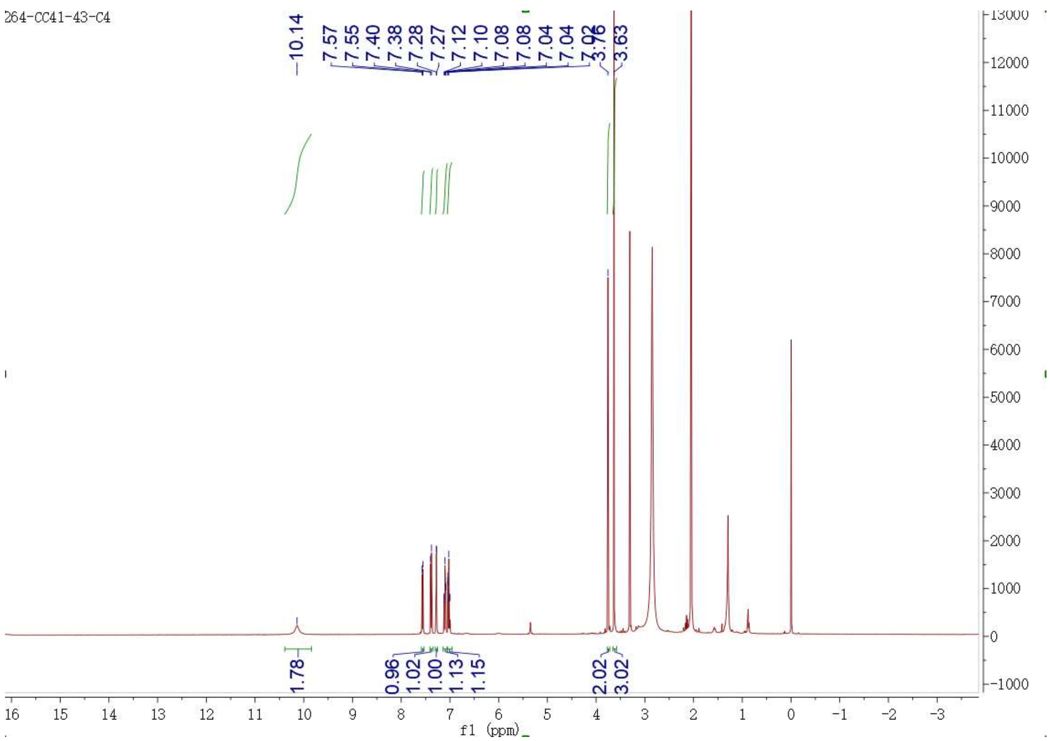

Figure S12. ^{13}C NMR spectrum of methyl Indol-3-ylacetate (**3**) in Acetone-*d*6 (400 MHz).

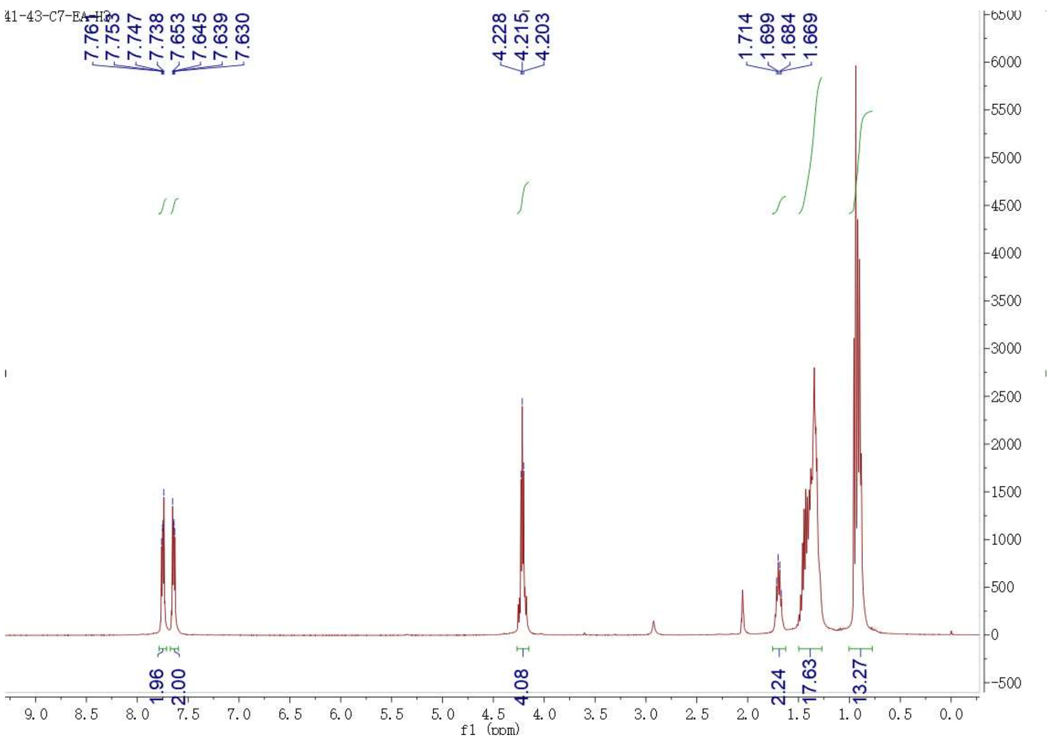

Figure S13. ^{1}H NMR spectrum of bis-(2-ethylhexyl) phthalate (**4**) in Acetone-*d*6 (400 MHz).

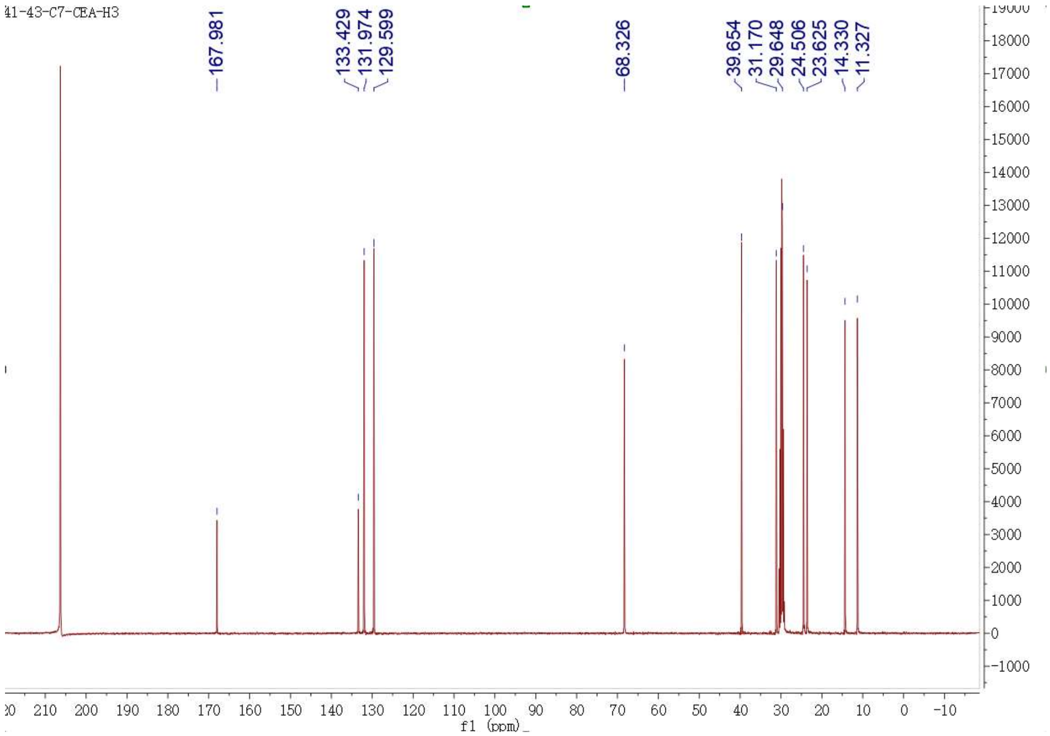

Figure S14. ^{13}C NMR spectrum of bis-(2-ethylhexyl) phthalate (**4**) in Acetone-*d*6 (400 MHz).

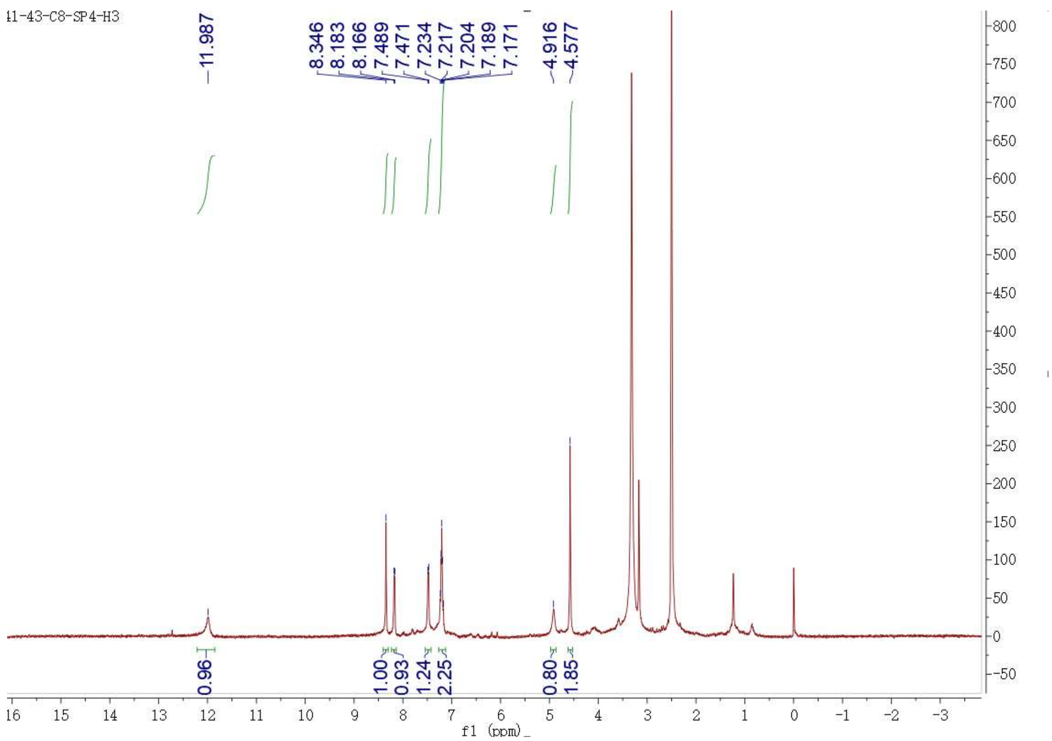

Figure S15. ^{1}H NMR spectrum of 3-(hydroxyl-acetyl)-1H-indole (**5**) in DMSO-*d*6 (500 MHz).

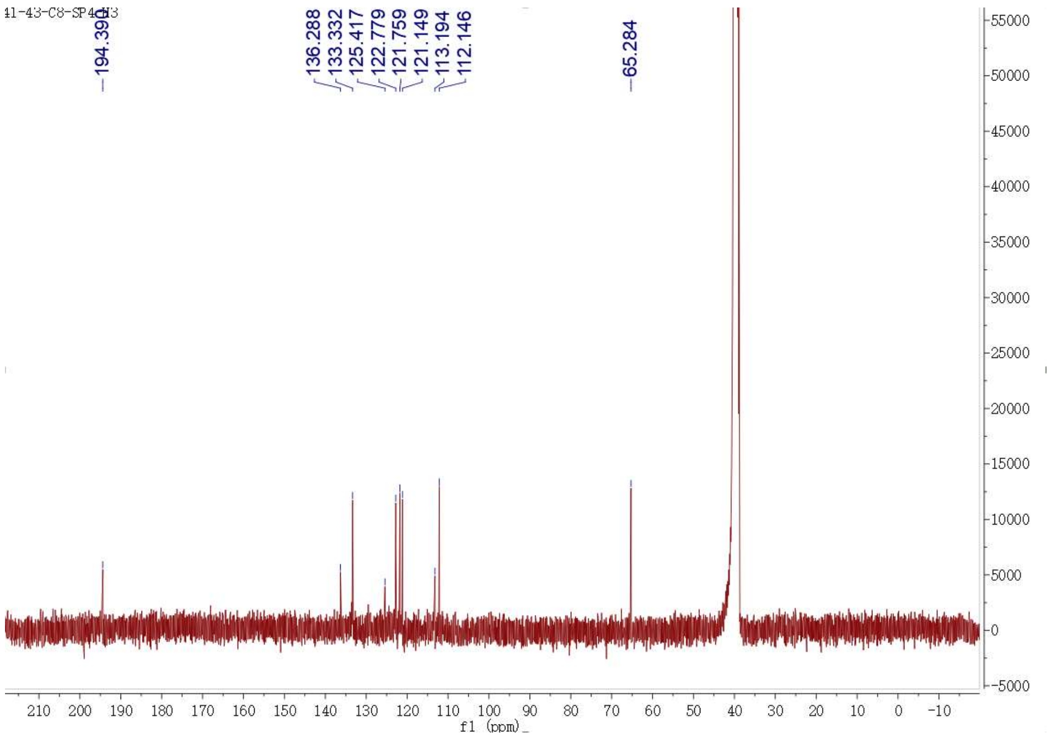

Figure S16. ^{13}C NMR spectrum of 3-(hydroxyl-acetyl)-1H-indole (**5**) in DMSO-*d*6 (500 MHz).

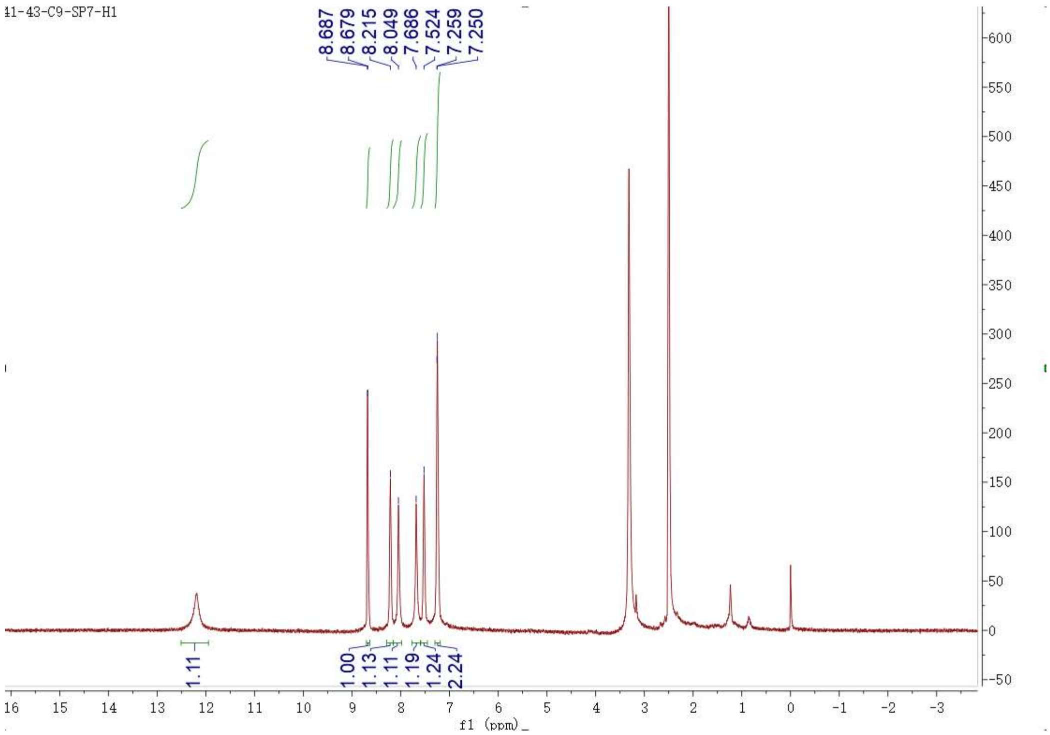

Figure S17. ^{1}H NMR spectrum of 2-(1H-indol-3-yl)-2-oxoacetamide (**6**) in DMSO-*d*6 (500 MHz).

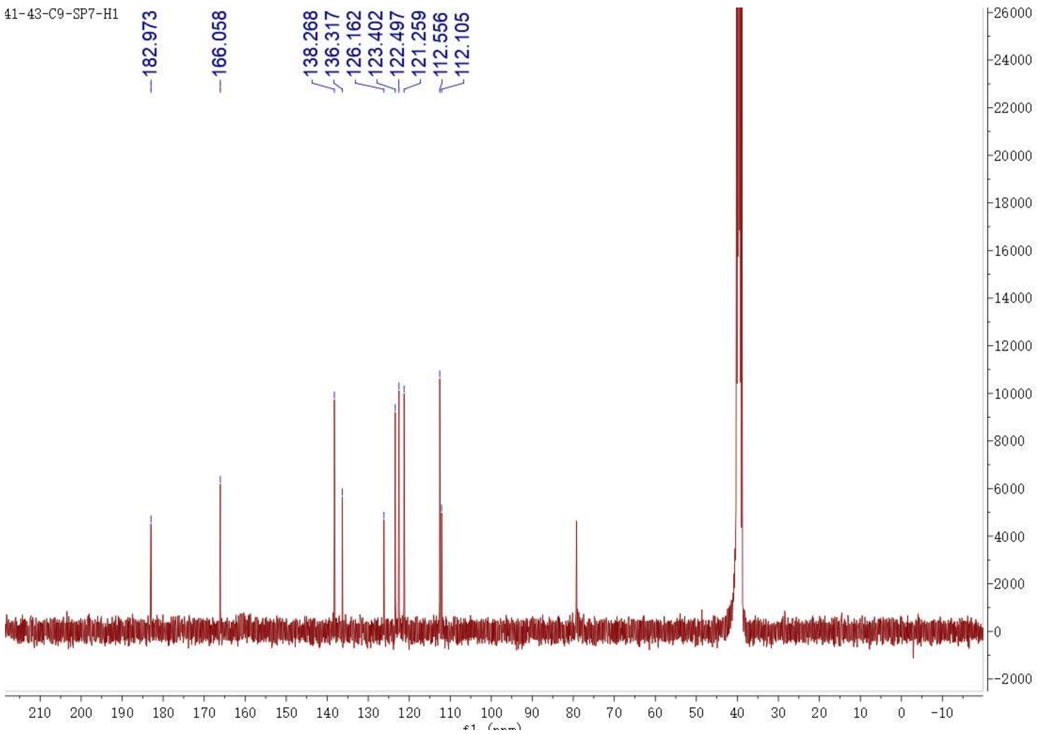

Figure S18. ^{13}C NMR spectrum of 2-(1H-indol-3-yl)-2-oxoacetamide (**6**) in DMSO-*d*6 (500 MHz).

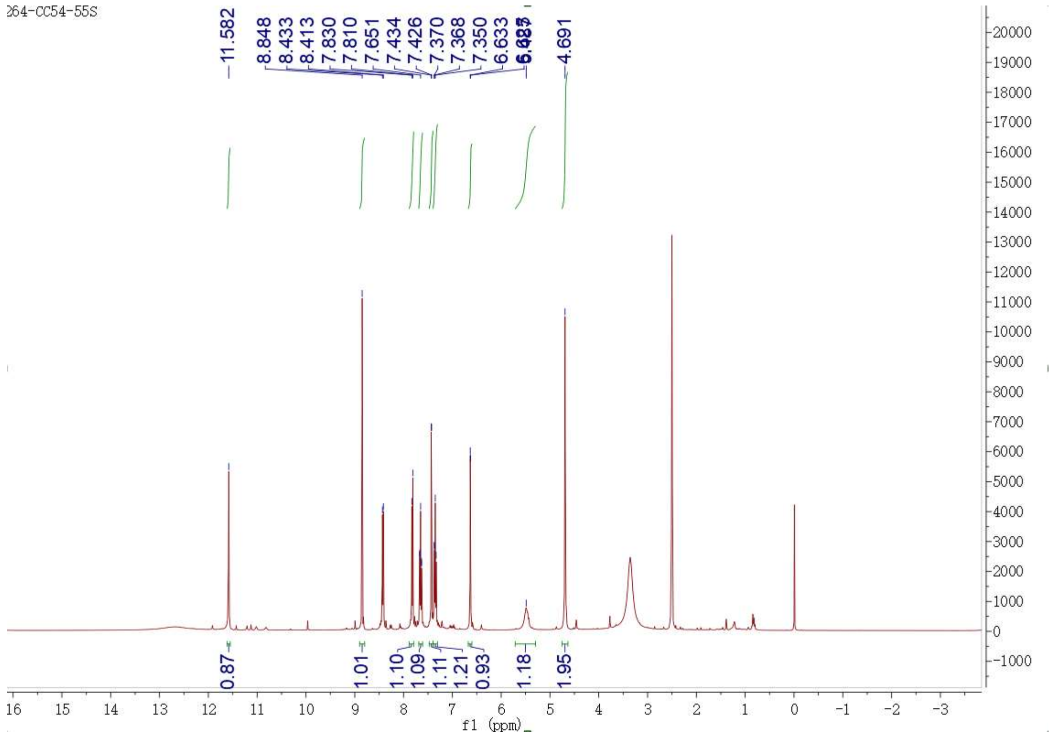

Figure S19. ^{1}H NMR spectrum of flazine (**7**) in DMSO-*d*6 (400 MHz).

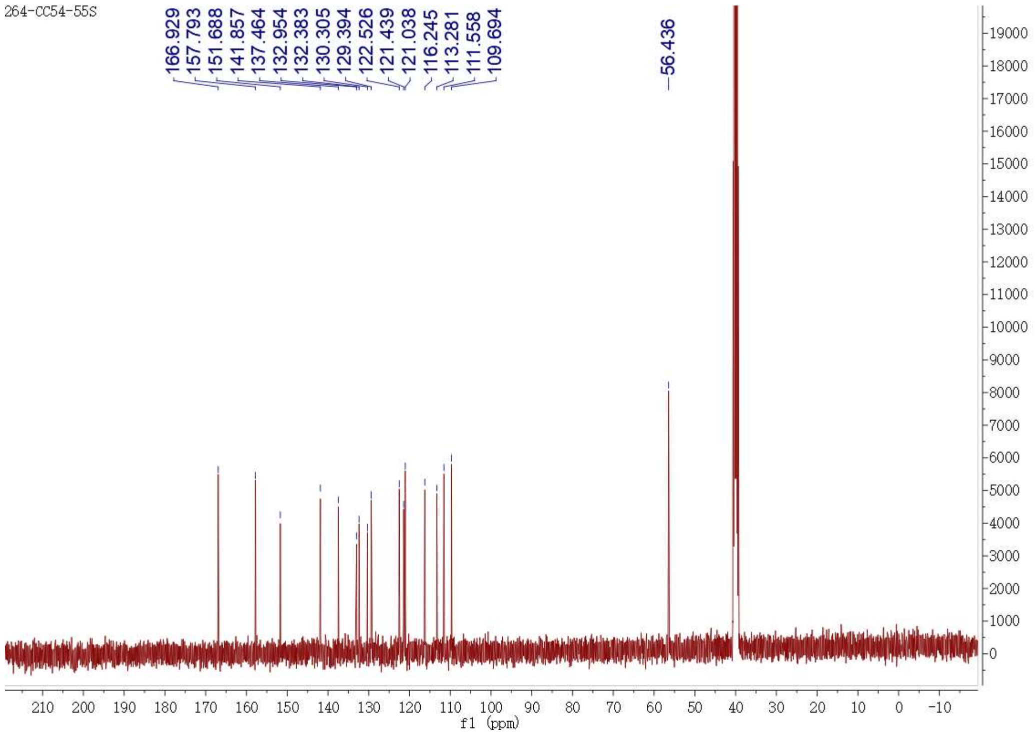

Figure S20. ^{13}C NMR spectrum of flazine (**7**) in DMSO-*d*6 (400 MHz).

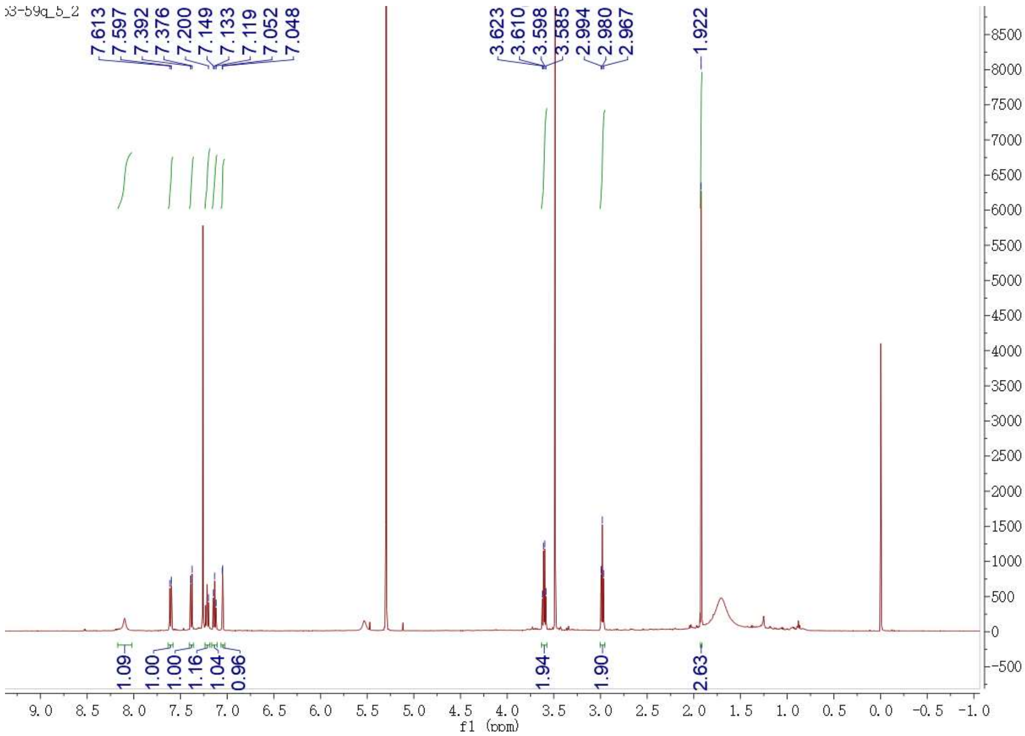

Figure S21. ^{1}H NMR spectrum of N-acetyltryptamine (**8**) in $CDCl_3$ (500 MHz).

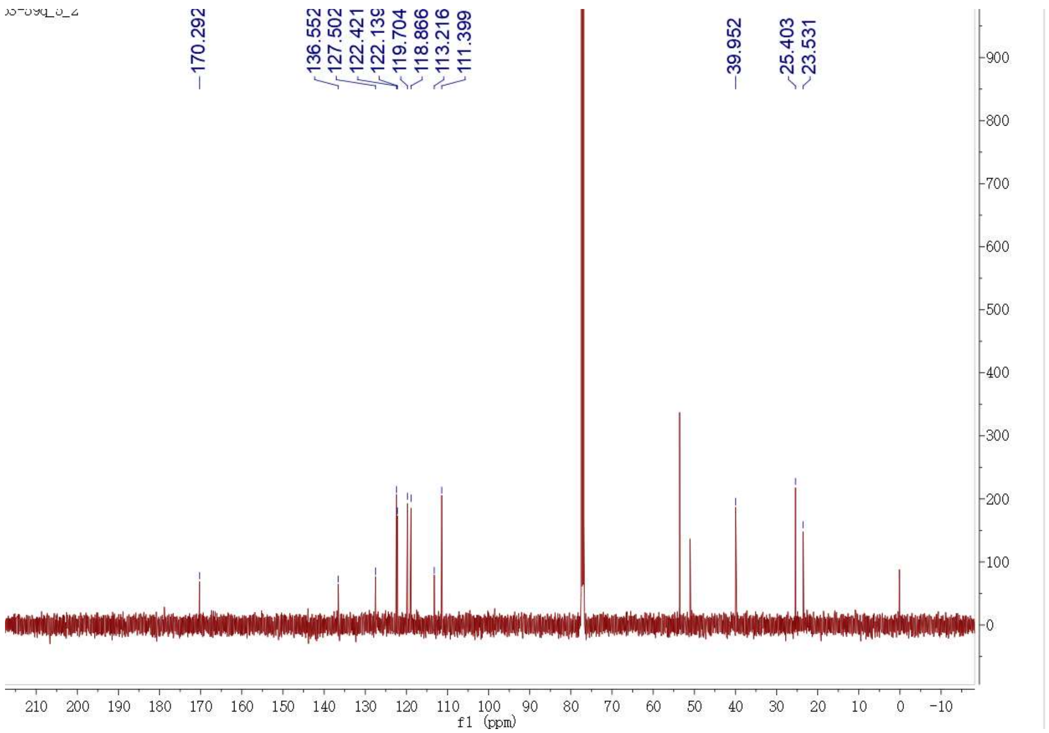

Figure S22. ^{13}C NMR spectrum of N-acetyltryptamine (**8**) in $CDCl_3$ (500 MHz).

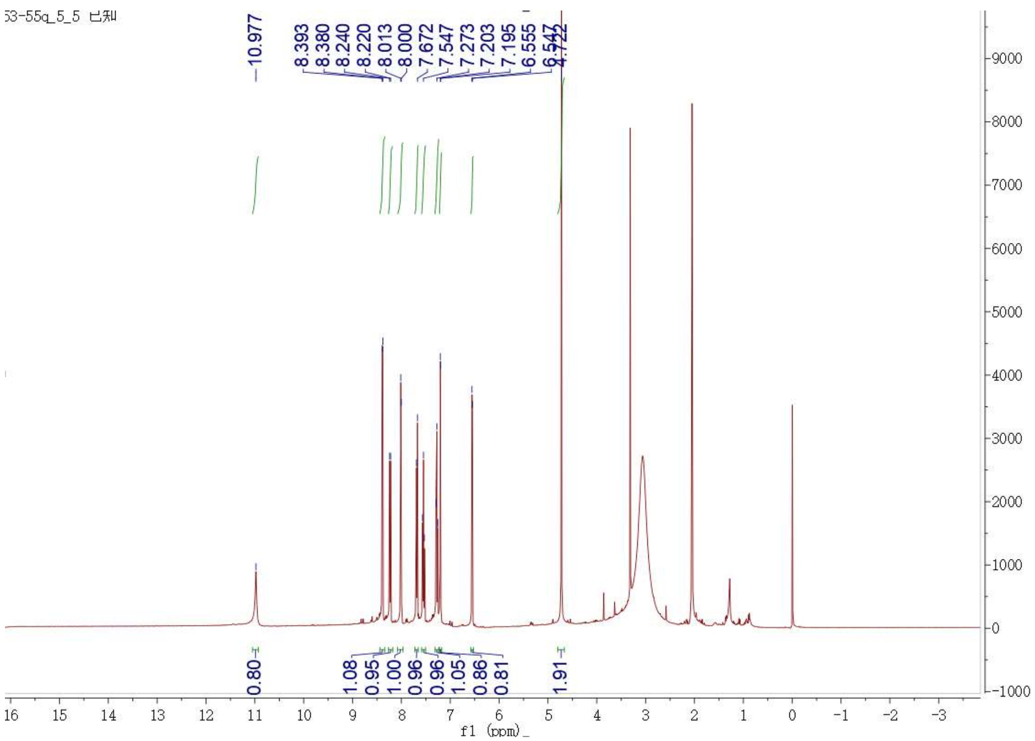

Figure S23. ^{1}H NMR spectrum of perlolyrine (**9**) in Acetone-*d*6 (400 MHz).

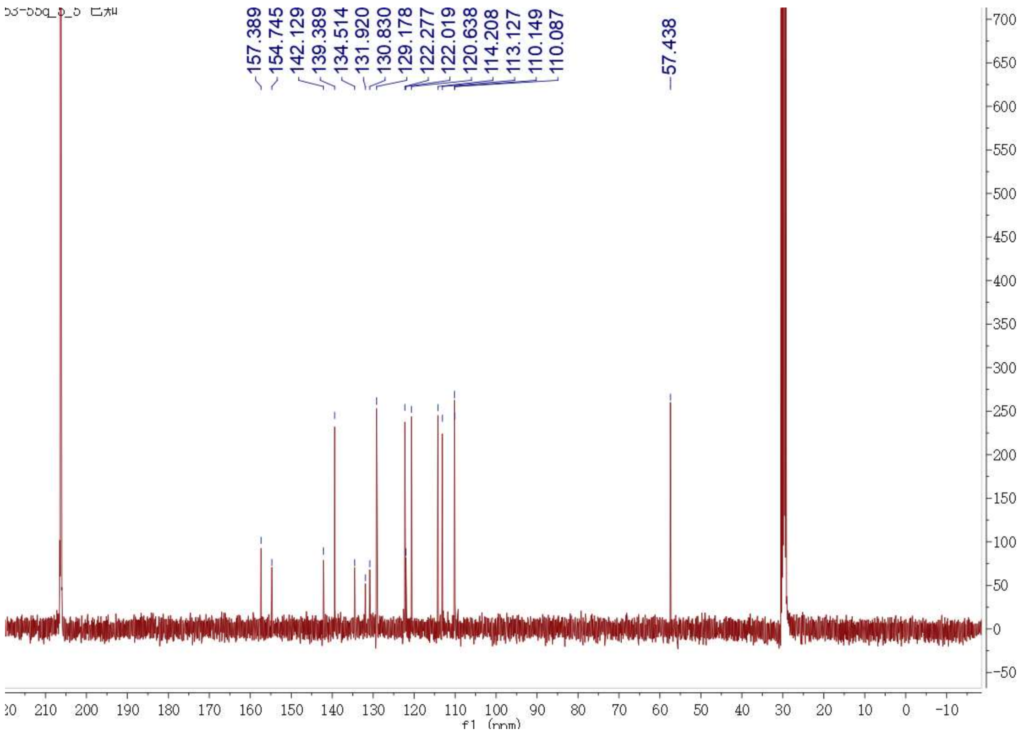

Figure S24. ^{13}C NMR spectrum of perlolyrine (**9**) in Acetone-*d*6 (400 MHz).

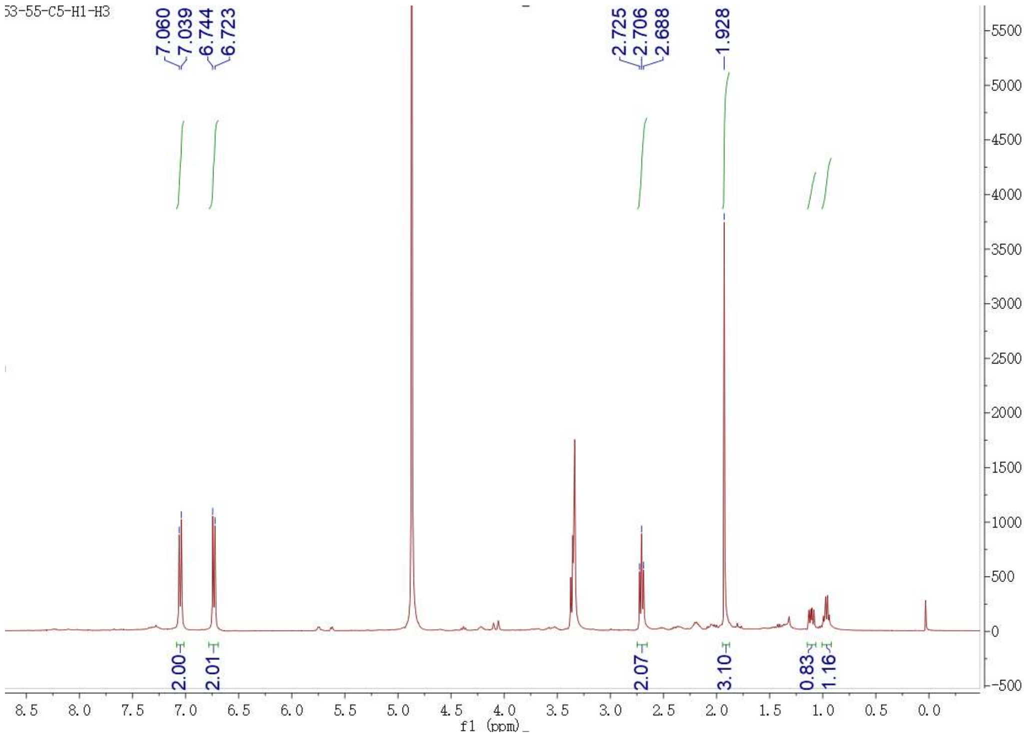

Figure S25. ^{1}H NMR spectrum of N-acetyltyramine (**10**) in MeOD (400 MHz).

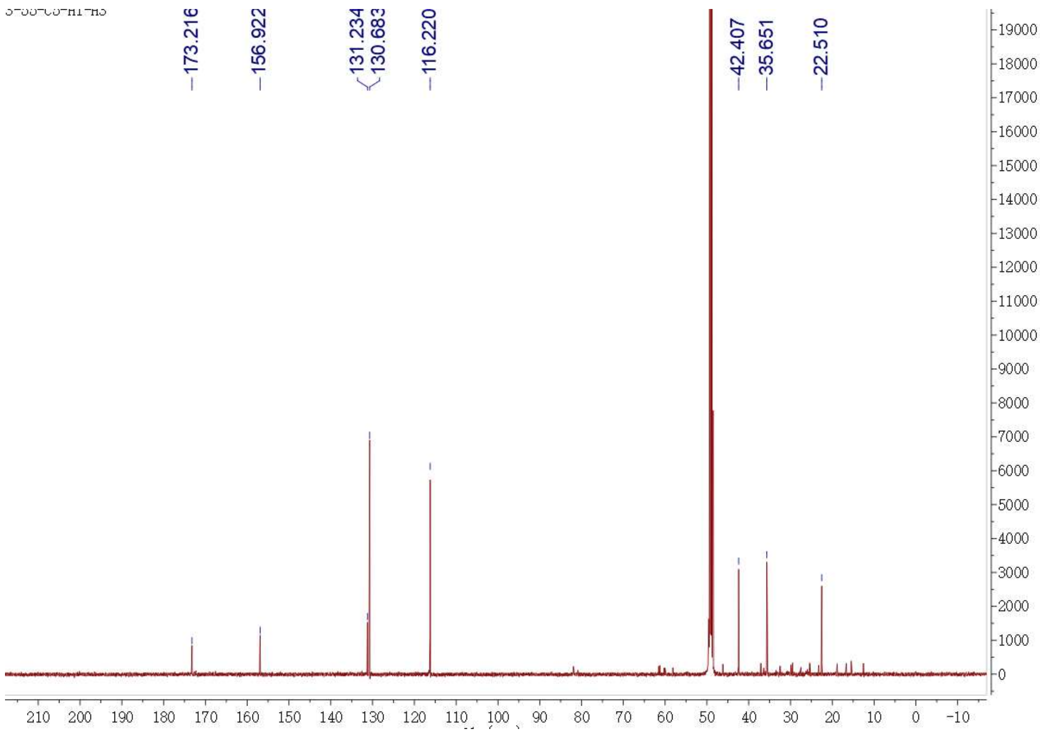

Figure S26. ^{13}C NMR spectrum of N-acetyltyramine (**10**) in MeOD (400 MHz).

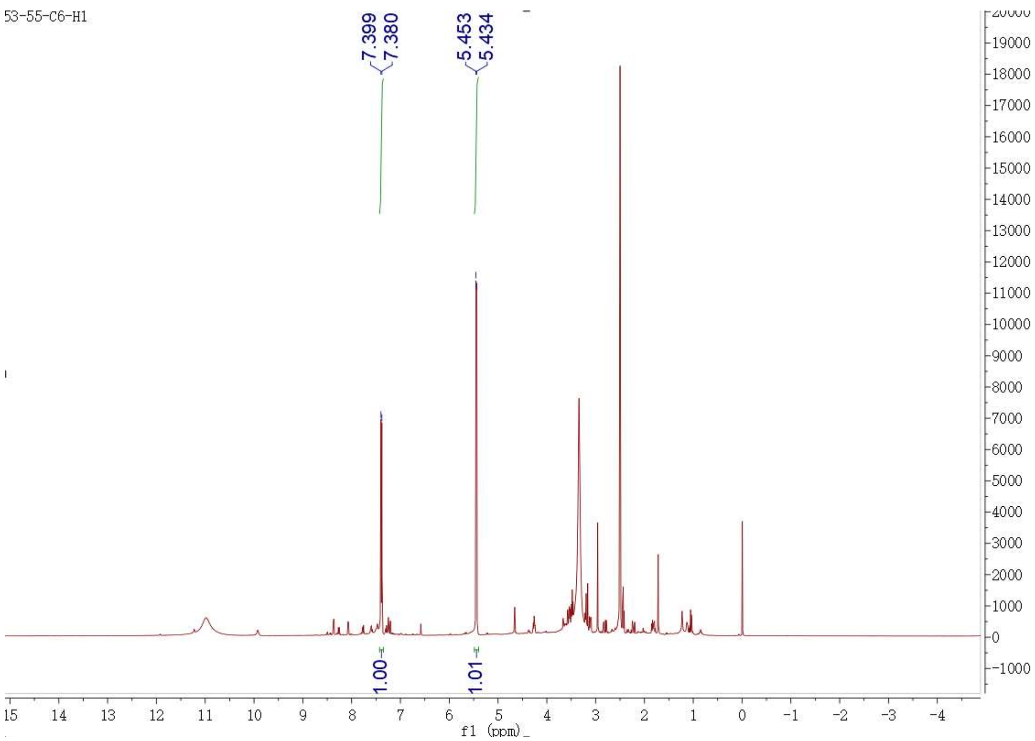

Figure S27. ^{1}H NMR spectrum of uracil (**11**) in DMSO (400 MHz).

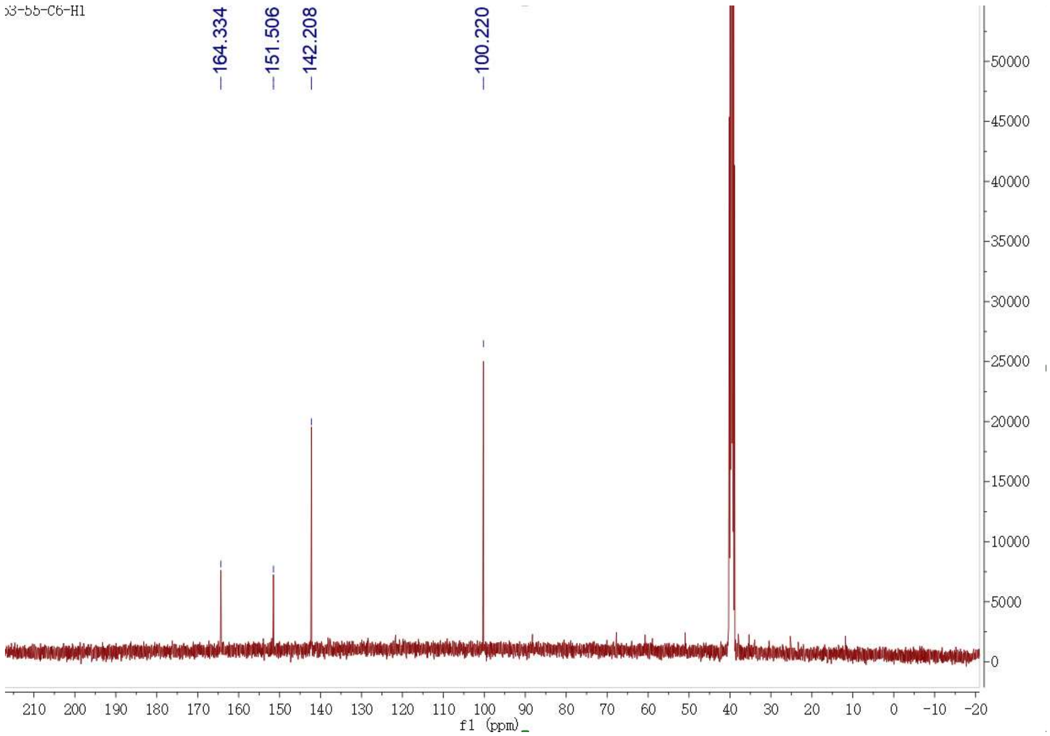

Figure S28. ^{13}C NMR spectrum of uracil (**11**) in DMSO (400 MHz).

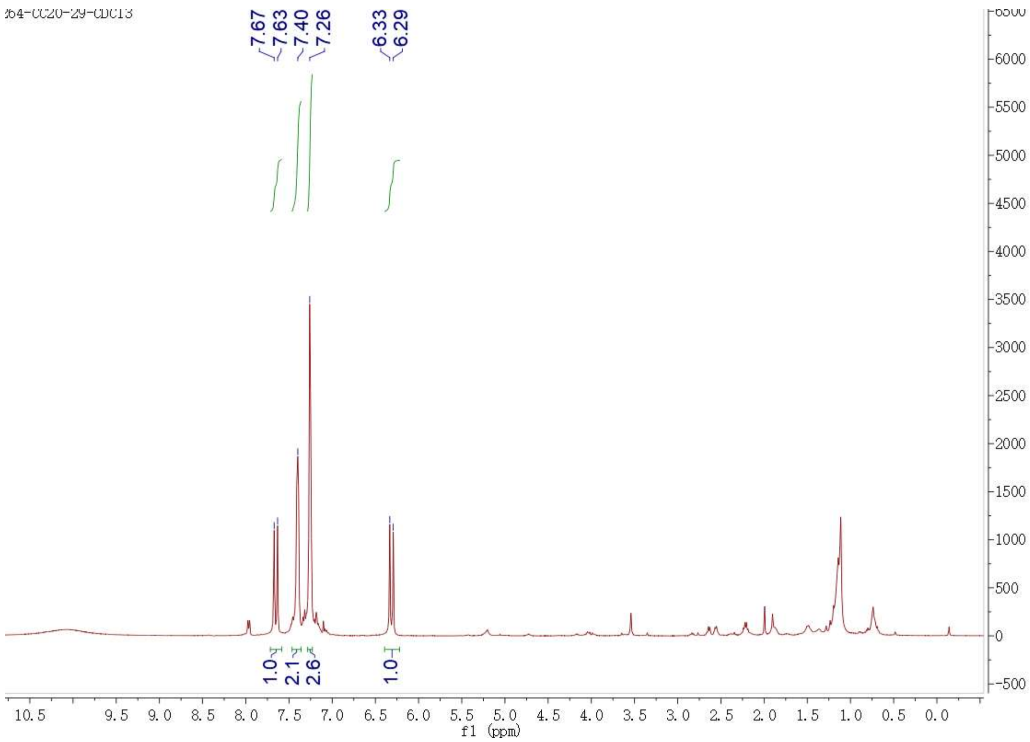

Figure S29. ^{1}H NMR spectrum of cinnamic acid (**12**) in $CDCl_3$ (400 MHz).

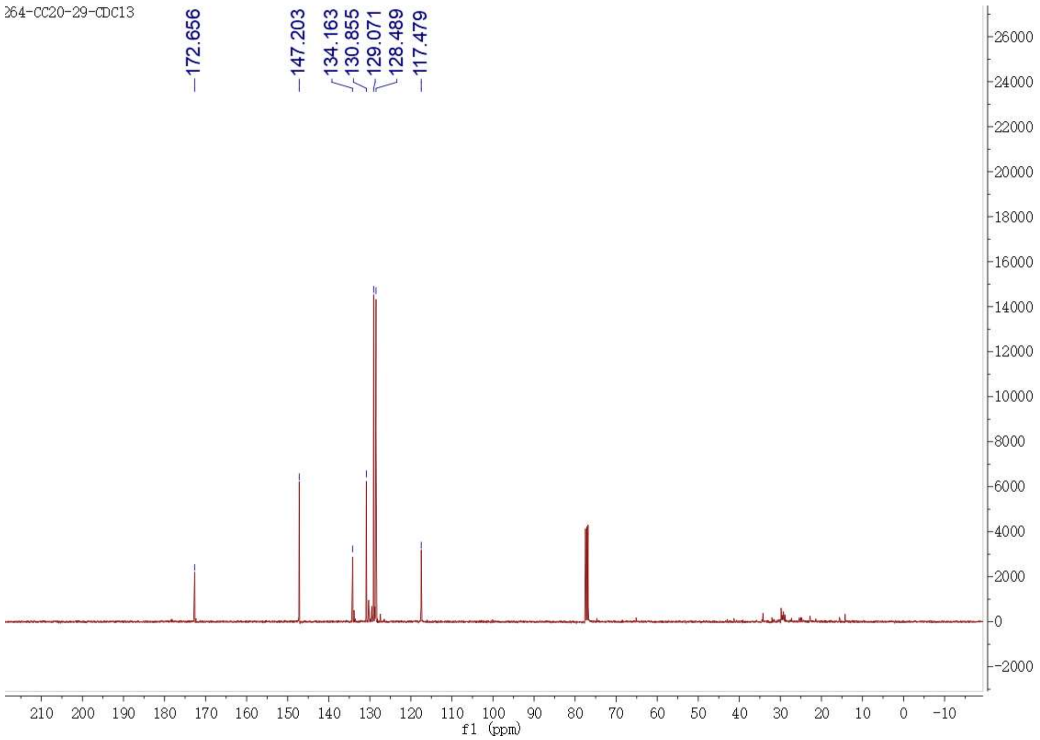

Figure S30. ^{13}C NMR spectrum of cinnamic acid (**12**) in $CDCl_3$ (400 MHz).
